# Cut or bind? Antigen-specific processing mechanisms define CD4^+^ T cell immunodominant epitopes for SARS-CoV-2 S and N proteins

**DOI:** 10.1186/s13073-025-01577-8

**Published:** 2025-11-26

**Authors:** Miguel Álvaro-Benito, Esam T. Abualrous, Holger Lingel, Stefan Meltendorf, Jakob Holzapfel, Paula de Diego Valera, Jana Sticht, Benno Kuropka, Cecilia Clementi, Frank Kuppler, Monika C. Brunner-Weinzierl, Christian Freund

**Affiliations:** 1 Laboratory of Protein Biochemistry, Department of Biology, Chemistry and Pharmacy. Freie, Universität Berlin, Berlin, Germany; 2 Department of Immunology, Ophthalmology and ENT, Universidad Complutense de Madrid School of Medicine, Severo Ochoa, Madrid, 28040 Spain; 3 Lymphocyte Immunobiology, 12 de Octubre Health Research Institute (imas12), Madrid, Spain; 4 Department of Mathematics and Computer Science, Freie Universität Berlin, Berlin, Germany; 5 Department of Physics, Faculty of Science, Ain Shams University, Cairo, Egypt; 6 Department of Experimental Pediatrics, Medical Faculty, Otto-von-Gericke-University, Magdeburg, Germany; 7 Mass Spectrometry Core Facility (BioSupraMol), Freie Universität Berlin, Berlin, Germany; 8 Theoretical & Computational Biophysics, Institute for Physics, Freie Universität Berlin, Berlin, Germany

**Keywords:** Antigen processing, MHCII presentation, CD4 + T cell epitope, Immunodominance, Mechanistic epitope prediction, HLA diversity, Immunogenomics

## Abstract

**Background:**

CD4⁺ T cell responses are key to adaptive immunity, yet the mechanisms underlying peptide selection and immunodominance across MHC class II variants in humans remain poorly defined. Two non-mutually exclusive models — First Bind-then cut (FBtc) and First Cut-then bind (FCtb) — have been proposed to explain immunodominant peptide selection, but experimental evidence in humans is mostly limited to a single allotype (HLA-DRB1*01:01).

**Methods:**

To generalize processing mechanisms across DRB1 alleles we developed an integrative strategy combining in silico prediction and a reconstituted antigen processing system. The independent and combined outcome of both approaches was validated on curated SARS-CoV-2 epitope data (IEDB) for responses to the Spike and Nucleocapsid proteins across a panel of 11 DRB1 allotypes, covering over 90% of European Caucasian populations. Potential immunogenic regions identified by the combination of both methods enabled the design of minimalistic peptide pools whose performance was validated via flow cytometry and ELISpot assays in post-Covid19 and pre-pandemic donors. Mechanistic insights for the selection of immunodominant peptides were derived analyzing biophysical parameters and proteolysis of the model antigens.

**Results:**

Three prediction tools used showed limited concordance for some allotypes (< 5%), but their combined output for all allotypes considered revealed potential immunogenic hotspots in the model antigens. Complementary, the reconstituted in vitro system identified allotype-dependent and promiscuous peptide candidates. Minimal peptide pools designed from the overlap of both methods featured improved performance to identify IEDB entries and induced robust CD4⁺ T cell activation in post-COVID-19 donors. Mechanistic modeling classified most immunodominant peptides from the Spike protein as arising via FCtb while FBtc predominated for Nucleocapsid. Epitope selection pathways are therefore antigen-dependent defined by proteolytic resistance and solvent accessibility.

**Conclusions:**

We establish a scalable, genomics-informed framework for decoding CD4⁺ T cell immunodominance across diverse HLA contexts. Our findings reveal that antigen-intrinsic features govern the preferential processing pathway — FCtb for Spike and FBtc for Nucleocapsid — and validate the utility of minimal peptide pools for population-level immune-monitoring. These insights inform the design of personalized immunotherapies and broadly effective vaccines.

**Supplementary Information:**

The online version contains supplementary material available at 10.1186/s13073-025-01577-8.

## Background

Human CD4^+^ T cell responses to pathogens are driven by a very specific subset of peptides selected from all available candidates in an antigen, a phenomenon known as immunodominance [1] — *Viz.* the preferential and recurrent recognition of peptides presented by Major Histocompatibility Complex (MHCII), also known as Human Leukocyte Antigens of class II (HLAII). Understanding and predicting immunodominance holds critical significance for immunologists but also for precision medicine provided the growing interest in personalized vaccines and immunotherapies [2]. The concept of T cell immunodominance was coined upon recall experiments with peptides following immunization of syngeneic mouse strains with model antigens, enabling the definition of binding motifs specific to MHCII molecules [1]. Binding motifs were regarded as the key to score immunodominance motivating binding predictions grounded on sequence searches. However, follow up studies demonstrated that the selection of peptides featuring immunodominant responses implies antigen-intrinsic properties (e.g., position of the epitope in the antigen), specific molecular mechanisms of antigen processing (e.g., peptide editing or proteases), the combination of allotypes expressed by the host, the frequency of T cell clonotypes and even exposure to similar antigens [3, 4] (Additional File 1: Box 1).

Immunodominance against new pathogens is a multifactorial phenomenon largely influenced by the selective presentation of peptides by MHCII at the cellular level. The canonical pathway of antigen presentation, involving endosomal processing, was considered responsible for immunodominant peptide selection [5]. However, alternative processing pathways have proven a considerable impact in shaping in vivo immunodominance [6]. Two molecular mechanisms explain peptide selection for MHCII presentation of pathogen-derived immunodominant epitopes: (i) the First Bind-then cut (FBtc) in which flexible and/or solvent exposed regions bind to MHCIIs and remain protected from degradation [7, 8], and (ii) the First Cut-then bind (FCtb), in which proteolysis yields peptides that are subsequently captured [9]. Although computational epitope prediction tools have attempted to incorporate aspects of these mechanistic constraints [10–12], no model has yet been fully validated to account for the diversity of processing routes and mechanisms resulting from human MHCII polymorphisms.

One of the major challenges when assessing immunodominant responses in humans is the genetic and functional diversity of MHCII allotypes, both within individuals and across populations. Unlike inbred mouse strains, humans possess a remarkable diversity of MHCII allotypes. Efforts aiming at identifying immunodominant peptides in humans focus almost exclusively on the kinetic stability of peptide-MHCII complexes (pMHCII) for single allotypes offering limited population-wide insights [9, 13]. An alternative, more pragmatic framework considers immunodominance at the haplotype level, emphasizing the collective peptide-binding capacity of MHCII allele combinations [14]. In this context, peptides derived from regions enriched in MHCII supertype binding motifs, and conserved sequence features from known epitopes, qualify as more likely to generate immunodominant responses [15]. The assessment of regions enriched for 7 MHCII supertypes along with the use of overlapping peptide pools underpins population-scale epitope prediction strategies with an estimated coverage of up to 50% of the total immune response for any antigen [16].

Here, we set out to uncover mechanistic and population-level determinants of CD4⁺ T cell immunodominance using the SARS-CoV-2 Nucleocapsid and Spike proteins as model antigens over a panel of representative MHCII allotypes. Integrating epitope predictions from state-of-the-art computational tools [10–12] with a reconstituted in vitro antigen processing system [17, 18], we defined minimalistic peptide pools with broad MHC coverage validating an improved performance of this pipeline. Systematic analysis of antigen processing constraints revealed distinct peptide selection mechanisms associated with antigen-specific features. Our work reinforces the multifactorial nature of immunodominance and highlights the importance of integrating genomic, structural, and processing-level data to build accurate, population-relevant epitope prediction models. Together, we provide a solid foundation for the systematic testing of the impact of antigenic features for the selection of immunodominant epitopes, essential for improving prediction tools.

## Methods

### In silico candidate epitope prediction

The SARS-CoV-2 genome sequence was obtained from the NCBI database https://www.ncbi.nlm.nih.gov/nuccore/1798174254. We extracted the sequences of the proteins orf1ab, S, orf3a, E, M, orf6, orf7a, orf7Bb, orf8, N, and orf10 based on the reference genome and corresponding to those reference in the main text. We used a sliding window size of fifteen amino acids and a step of one amino acid for the following analysis (9591 peptides). Potential SARS-CoV-2 epitopes were identified using a novel selection workflow based on the integration of prediction algorithms for peptide-MHC class II binding and immunogenicity. The peptide-MHC class II binding/presentation prediction was performed using three different algorithms, netmhcIIpan [12], MARIA [10], and NeonMHCII [11], with percentile score cut-off of 10%. Immunogeicity scores used as flagging criteria were determined using the IEDB tool [15] applying an immunogenicity score cutoff value of 50%, yielding 164 immunogenic peptides. Potential SARS-CoV-2-derived epitopes were identified as the top-ranked overlapping candidates for each allotype of the eleven MHC class II allotypes. Next, allele-specific lists of peptides with a minimum length of 15 residues were defined for each SARS-CoV-2 protein.

### Peptides and viral antigens

Peptides were purchased from GL Biochem (Shanghai) Ltd (10 mg purity > 95%). Lyophilized peptides were diluted at a final concentration of 10mM in DMSO and subsequently diluted in PBS. When necessary, the pH was adjusted to 7.4. Nucleocapsid and Spike Glycoproteins expressed in HEK cells were purchased from Sino biological (Cat Numbers. 40588-V08B for Nucleocapsid and 40589-V08B1 for Spike) as C-terminal His-tagged. Lyophilized proteins were reconstituted according to the manufacturer specifications.

### Protein methods

MHC proteins (HLA-DRs and HLA-DM) were expressed as previously described [19]. Briefly, HLA-DR cDNAs are cloned into the pFastBacDual vector and include Leu-Zippers in their C-termini as well as a sequence encoding for the CLIP peptide followed by Thrombin cleavage site and a G4S linker in the N-termini of the DRB1 polypeptides. Furthermore, the DRA polypeptide encodes a Biotin Acceptor Sequence at the C-termini of the corresponding Leu-Zipper. Expression was achieved by infection of Sf9 cells at an MOI of 5 and harvesting the cells after 4 days. Protein purification was achieved by immunoaffinity chromatography using a L243-FF-Sepharose resin casted in house. In all cases HLA-DR proteins were cleaved with Thrombin and gel-filtrated using a Sephadex S200. For the reconstituted in vitro system, these proteins were also cleaved with V8 protease to remove the Leu-Zippers. HLA-DM cDNAs are cloned into pFastBacDual and include a Flag-Tag in the C-termini of HLA-DMA chain. Purification in this case was achieved using an immunoaffinity M2-Sepharose resin, protein was eluted using Glycine pH 3.5. After dialysis, the protein was concentrated (Vivaspin MWCO 10 kDa) and gel filtrated (Sephadex S200).

### Peptide binding affinity measurements

Peptide binding affinities to selected HLA-DR molecules were determined by competition experiments using fluorescently labelled reporter peptides. Reporter peptide binding signal was measured by FP. HLA-DR molecules expressed in insect cells were thrombin cleaved to facilitate peptide exchange. Competition experiments were set by adding 100 nM HLA-DR, 100 nM reporter peptide (CLIP-FITC for DRB1*07:01 or MBP-FITC for DRB1*03:01) and tittered concentrations of the corresponding peptide in 50mM Citrate Phosphate buffer containing 150mM NaCl at pH 5.3. Each reaction was measured after 12 h incubation at 37° C, and the corresponding IC50 values for each peptide were retrieved by fitting a sigmoidal function to the obtained data points.

### In vitro reconstituted antigen processing system

The previously described cell-free reconstituted in vitro system [17] was modified according to the specific needs of the experiments [18]. HLA molecules (200 nM) together with the candidate antigens (500 nM) and the HLA-DM (50 M) were incubated for 2 h at 37° C in citrate phosphate 50 mM pH 5.2 in the presence of 150 mM NaCl. Cathepsins were added to reaction mixtures after incubation with L-Cysteine (6 mM) and EDTA (5 mM). The final reaction mixture was incubated at 37° C for 2 to 5 h. Afterwards the pH was adjusted to 7.5, and Iodoacetamide was added (25 mM). Immunoprecipitation (IP) of the pMHCII complexes was performed using L243 covalently linked to Fast Flow sepharose. Peptides were eluted from purified MHCII adding Tri-Fluoracetic Acid (TFA) 0.1% to th samples. Peptides are separated from the MHCII molecules by using Vivaspin filters (10 kDa MWCO). Cathepsin B (Enzo), H (Enzo) at a molar ratio of 1:250, and S (Sigma) at a molar ratio of 1:500, respectively were used in these experiments.

### Proteolytic degradation of antigens

Spike and Nucleocapsid proteins were incubated in the presence of cathepsins in the molar ratios indicated above. Reactions were performed at 37 °C citrate phosphate 50 mM pH 5.2 in the presence of 150 mM NaCl and stopped at t = 0, and t = 3 h, by adding Iodoacetamide, immediate transfer of the samples to ice. The pH was then adjusted to 7.5 by adding Tris-HCl 1 M pH8.0. Samples were splitted, and used for SDS-PAGE analysis, Western blotting and peptide identification by MS. For MS analysis, samples were dried in a SpedVac and treated as described below.

### LC-MS measurements

All samples were initially cleaned up by reverse phase C18 enrichment. The eluates were dried in a SpeedVac, and peptides were reconstituted in 20 ml of H_2_O containing acetonitrile (4%), and TFA (0.05%). 6 ml of these mixtures were analyzed using a reverse-phase capillary system (Ultimate 3000 nanoLC) connected to an orbitrap connected to a Q Exactive HF mass spectrometer (Thermo Fisher Scientific). Samples were injected and concentrated on a trap column (PepMap100 C18, 3 μm, 100 Å, 75 μm i.d. × 2 cm, Thermo Fisher Scientific) equilibrated with 0.05% Formic Acid (FA) in water. After switching the trap column inline, LC separation was performed on a capillary column (Acclaim PepMap100 C18, 2 μm, 100 Å, 75 μm i.d. × 25 cm, Thermo Fisher Scientific) at an eluent flow rate of 300 nL/min. Mobile phase A contained 0.1% FA in water, and mobile phase B contained 0.1% FA in 80% acetonitrile/20% water. The column was pre-equilibrated with 5% mobile phase B followed by a linear increase of 5–44% mobile phase B in 70 min. Mass spectra were acquired in a data-dependent mode utilizing a single MS survey scan (*m*/*z* 350–1,650) with a resolution of 60,000, and MS/MS scans of the 15 most intense precursor ions with a resolution of 15,000. The dynamic exclusion time was set to 20 s and automatic gain control was set to 3 × 10^6^ and 1 × 10^5^ for MS and MS/MS scans, respectively.

### Mass spectrometry data processing

MaxQuant (v2.0.3.0) with implemented Andromeda peptide search engine was used for analyzing the raw MS and MS/MS data. All searches were done on the basis of unspecific protease cleavage, main ion search tolerance of 10 ppm and MSMS tolerance search of 50 ppm and enabling the feature “match between runs”. The reconstituted in vitro antigen processing samples were searched against a database containing the sequences of all SARS-CoV-2 proteins (note that Spike and Nucleocapsid were substituted for the sequences of the recombinant ones), cathepsins, MHCII and all reviewed *Spodoptera frugiperda* proteins (Uniprot, access in March 2020) as an internal control. The database used for the cathepsins digestion experiments included only the protein antigen sequence used, and the corresponding sequences of the cathepsins. In both cases a False Discovery Rate (FDR) of 0.01 (1%) was used as well as a decoy database search. All identifications with a FDR higher than 0.01, reverse identifications and contaminants (identified by MaxQuant) were removed for data analysis. Each set of experiments was analyzed together, treating technical and biological replicates as independent samples.

All MS raw files from the reconstituted in vitro antigen processing experiments were processed as previously reported in [18]. In brief, allotype-specific subset identifications from the evidence file were submitted to the plateau webserver using the same database used for the peptide identification. Each of the consensus peptides identified was then used to determine replication and retrieve a MS1 relative intensity. These relative intensities were averaged throughout the different replicates, and unless otherwise indicated only peptides found in at least 2 technical replicates out of 2 independent experiments were considered. For the proteolytic degradation experiments we took the spectral counts for each peptide identified from the corresponding peptide.txt files. Thus, the number of times that every residue could be mapped to the protein´s sequence.

### Processing of donor samples: PBMC isolation and fractionation

PBMCs isolated from blood samples of either healthy individuals or donors recovered from SARS-CoV-2 infection were isolated via density-gradient sedimentation. Thawed PBMCs were first resuspended in complete medium and counting of living cells was performed using trypan blue in a cell counting chamber. When stated PBMCs were used directly in specific experiments, and for others specifically isolated cellular fractions were used. In these cases, cells were isolated by magnetic cell separation using CD14 microbeads for monocytes, and subsequently CD4 microbeads for T helper cells (all Miltenyi Biotec, Bergisch Gladbach, Germany). The homogeneity of the cell preparations was controlled by flow cytometry. For flow cytometric analysis a total of 1 × 10^5^ PBMCs per well were seeded on 96-well plates, cultured in X-VIVO 15 medium (Lonza, Basel, Switzerland) supplemented with 4% human AB plasma (Innovative Research, Novi, MI, USA), and provided with the corresponding peptide pools (17.5 ng/ml per peptide).

### ELISpot

Dual secretion of IFNγ and IL-10 was determined using the enzymatic Human IFNγ/IL-10 Double-Color ELISPot Kit (Cellular Technology, Shaker Hieghts, OH, USA) and using pre-isolated CD14^+^ monocytes and CD4^+^ T cells. 5 × 10^4^ APC were seeded together with 1 × 10^5^ CD4^+^ T cells in the presence of relevant peptides at 2.5 ng/ul diluted in X-VIVO 15 medium (Lonza, Basel, Switzerland) supplemented with 4% human AB plasma (Innovative Research, Novi, MI, USA) in 96-well plates. The cells were then pre-incubated for 48 h, washed, transferred to an ELISpot plate and further incubated for 60 h. The secreted cytokines were determined according to the manufacturer’s instructions and counting of spots place on an ImmunoSpot analyzer (Cellular Technology). Dual secretion of cytokines was determined by overlapping the corresponding signals (IFNγ-red and IL-10-blue). The extent of the response is directly correlated to the surface area covered by each signal, in this case determined for each colony counted.

### Antibodies and reagents used in cell culture experiments

The following antibodies were purchased from the stated vendors and used according to the manufacturer specifications: for western-blots Rabit anti-6HisTag (Abcam ab1187). In flow cytometry experiments we used: anti-CD4, anti-CD137, anti-CD319 (all Miltenyi Biotec), anti-CD3, anti-PD-1, anti-IL-2, anti-TNFα, anti-IFNγ (all Biolegend). And in case of Elispots we considered: anti-IFNγ (capture and FITC-labelled detection antibodies), anti-IL-10 (capture and biotinylated detection antibodies), anti-FITC HRP (all Cellular Technology).

### Flow cytometry

To accumulate cytokines, cells were treated for 4 h with 5 mg/ml Brefeldin A (Merck, Darmstadt, Germany) after 140 h incubation time. Further, cells were briefly reactivated by addition of 10 ng/ml PMA and 1 mg/ml ionomycin (all Merck) for 1 h prior to flow cytometric analysis. Cells were then harvested, Fc-receptors blocked (FcR Blocking reagent, Miltenyi) and stained for extracellular markers (CD3, CD4, CD137, PD-1, and CD319), subsequently fixed with 2% paraformaldehyde (Morphisto, Offenbach am Main, Germany), permeabilized with 0.5% saponine (Merck), and stained for intracellular cytokines (IFNγ, IL-2, and TNFα). Samples were acquired on a FACSCanto II flow cytometer with FACS-Diva software (v10, BD Bioscience).

### IEDB data retrieval and analysis

We opted for a scheme, where epitopes flagged as tetramers and with affinity values below a conventional threshold (Aff < 1000nM) were sufficient to define HLA restrictions of a ligand, similar to a recent publication [20]. In contrast to this conservative approach, we considered entries eliciting recurrent responses (RF > 0.5, and *n* > 10, named here HF_TcAs here) where the restriction element was unknown. Our DRB1* panel has a decent coverage throughout the individuals in these studies, hence these allotypes are potential restricting elements of those HF_TcAs. Exemplarily, 80% o the individuals recruited in one of the most comprehensive studies validating immunodominance to SARS-CoV-2 expresses at least one of the allotypes in our DRB1 panel [21]. Likewise, if we take into account all epitopes described in TcAs data with associated HLA information, the estimated probability that one of our selected allotypes is the restricting element is higher than 0.5 in approximately 80% of the entries. This value reflects the phenotypic coverage for each individual entry of our panel according to those restrictions stated by these studies.

### Analysis of antigen processing rules and antigenic peptide features

Each protein residue was assigned a value for each of the following parameters: resistance to proteolytic degradation (Res_Prot), relative surface accessible solvent area (rel_sasa) and a pseudo-Sensitivity to endo-protease digestion. For rel_SASA we used the relative values calculated using the pdb PISA tool [22] using as input the structural models available for the Spike and the Nucleocapsid protein as of September 2021 provided by the Zhang lab and available at [23]. Res_Prot considers spectral counts for every residue. Pseudo endo-protease cleavage (sensitivity) was retrieved by analyzing proteolytic maps. We deemed residues to be resistant to proteolysis if they met two criteria: (i) it was identified in three or more reaction mixtures, and (ii) this criterion was met for at least seven consecutive amino acids. Note that this approach yields regions where endo- proteolysis has occurred and at the same time exo-proteases may have trimmed the peptide ends. For this analysis all available entries in the IEDB described as epitopes, ligands or T cell Assay related (TcAs) were equally coded as binary vectors (0: not present, 1: part of a hit) (Accessed Sept 2022). The sum of appearances of each residue as a Hit facilitates defining their relative frequency. If additional data was available, e.g. binding affinity measured experimentally, this information was used to subset those hits. Finally, each candidate epitope identified by the reconstituted antigen processing system was coded at the amino acid level considering the MS1 intensity (e.g. Spike_DRB1*01:01_n for candidate n derived from the Spike protein identified for DRB1*01:01). We worked with either normalized (to the maximum of each vector), count-based of hits or binary-based vectors, as stated in each case.

### Statistical analysis and model description

All analysis were carried out using R, version 4.2.1 over the RStudio suite unless otherwise stated.

Binary logistic regression models were generated based on the general expression:

$$\:logit\left(p\right)\sim\:({\beta\:}_{0}+{\beta\:}_{1}{x}_{1}+{\beta\:}_{2}{x}_{2}+\dots\:+{\beta\:}_{k}{x}_{k})$$

$$\:p\left({x}_{i}\left.Hit\right|{x}_{i}predictors\right)$$

In all cases response variables at the residue level were transformed to binary values for the different types of IEDB curated entries (Ligand, Tetramers and HF_TcAs). Either in silico predictors (the three or their sum) or experimental outputs were considered independently and used as explanatory variables ($\:{\beta\:}_{0}$ to $\:{\beta\:}_{n}$). Subsequently, each subset of predicted candidates from the in silico tools (ipEp), identified experimentally (epEp) or its combination (pEp: ipEp and epEp) were considered as single or combined explanatory variables. Goodness of fit and the performance of the models were estimated by calculating the Akaike Information Criterion (AIC), the Area Under the Curve (AUC) from Reiceiver Operator Curves (ROC). 10-fold cross-validation was used to assess the performance of the logistic regression models.

## Results

### Establishing a conceptual framework for studying mechanistic insights of CD4^+^ T cell immunodominant responses in humans

**Fig. 1:**
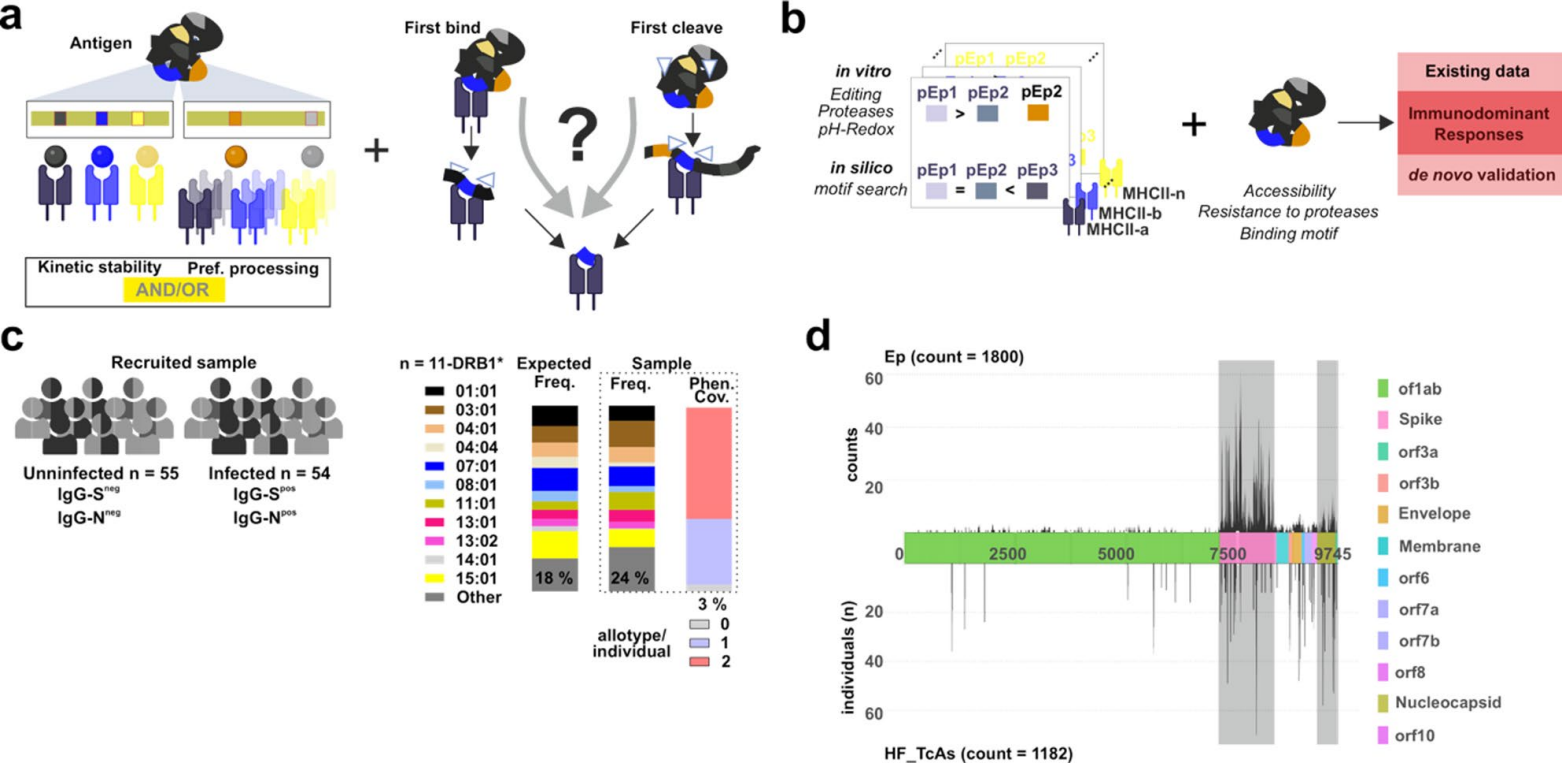
Conceptual and experimental framework to assess key features of immunodominant CD4^+^ T cell responses. **a**. Existing frameworks studying immunodominance are usually considered mutually exclusive. One of these views is based on kinetic stability (studies individual allotypes) and the other one considers on promiscuous binding (haplotype restriction capacity). These views are depicted over a model antigen with 5 potential epitopes (color coded). Depending on the definition of immunodominance, the focus is on peptides binding to one allotype (kinetic stability model), or the peptides bound by all allotypes in a haplotype (promiscuous binding model). Pathogen-derived immunodominant epitopes are selected through the First Bind-then cut or the First Cut-then bind mechanism. The contribution of each mechanism to immunodominant epitope selection is not yet clear (“?”). **b**. We leveraged on predictions from in silico tools, identifying binding motifs with the output of a reconstitution antigen processing system resembling cellular processing to define immunodominant epitopes. **c**. A sample of 109 individuals whose viral infection status was validated (55 uninfected and 54 SARS-CoV-2 infected) was considered to test the phenotypic coverage of our DRB1 panel (shown in the right side of the panel) and candidate epitopes. **d**. Overview of reported immune responses to SARS-CoV-2 antigens at the Immune Epitope Data Base (IEDB accessed Sept. 2022 [27]). The entire viral proteome is depicted in the middle part of the graph indicating amino acid positions and orfs. The count of epitope entries is shown in the upper part of the viral proteome scheme. The number of individuals reacting to peptides defined as High Frequent T cell Assay entries (HF_TcAs, tested in more than 10 individuals and yielding a response frequency higher than 50%) is shown in the lower part of the scheme. Spike and Nucleocapsid data is highlighted over a gray background

Pathogen-derived peptides triggering immunodominant responses bind with a high kinetic stability to one or various MHCII allotypes (Fig. 1a, left). Peptides triggering these responses are selected through either the FBtc [8, 13] or FCtb [9] mechanism (Fig. 1a, right). However, experimental evidence for the selection of immunodominant peptides through the abovementioned models in humans is restricted to a single allotype, DRB1*01:01. To determine whether these models generalize across other MHCII allotypes, we conceived expanding our study to a broader set of DRB1 allotypes [24].

We devised and developed an integrative strategy based on in silico tools and a reconstituted in vitro system recapitulating antigen processing (Fig. 1b). We selected a panel of 11 DRB1 allotypes providing a high phenotypic coverage throughout different populations (e.g., European 85 to 95% and African 55 to 70%) averaging 90% for European Caucasians [25]. Indeed, this panel reached up to 96% phenotypic coverage for our 109 donors (Fig. 1c, Additional File 1: Table S1) validating its applicability. The presence of IgG antibodies targeting the Spike protein allowed us classifying individuals as post-Covid19 (PC). Pre-pandemic samples (collected between September 2018 and 2019) were used as un-infected (HD) [26].

To guide our approach, we leveraged existing information of SARS-CoV-2 CD4^+^ T cell responses by retrieving all available datasets from the Immune Epitope Data Base (IEDB) as of September 2022 (Fig. 1d upper-part and Additional File 1: Fig S1a-e) [27]. Relevant studies for our work varied considerably in size (n of individuals tested), broadness (n of antigens tested) and methodology used for testing immunogenicity. For instance, the high number for T cell Assays (TcAs, *n* = 3437) contrasts with the low count of multimer-identified epitopes (Tet_TcAs, *n* = 149). Entries tested over 10 individuals and reaching Response Frequencies (RF) [28] higher than 0.5, are considered “High Frequency” TcAs (HF_TcAs, Fig. 1d lower part). The DRB1 panel reaches a phenotypic coverage on the individuals and datasets collected going between 60% and 95% (Addiional File 1: Table S2 and S3). Even though most SARS-CoV-2 proteins have proven to be immunogenic we decided to focus on the Spike and Nucleocapsid proteins as main targets of immune responses to viral infection using the reference sequences of the Wuhan-Hu-1 isolate (ref. seq accession number Genebank: 1798174254; Fig. 1d and zoom in Additional File 1: Fig S1f and g).

### In silico predictions of MHCII-binders/presented peptides highlights immunogenic regions

**Fig. 2:**
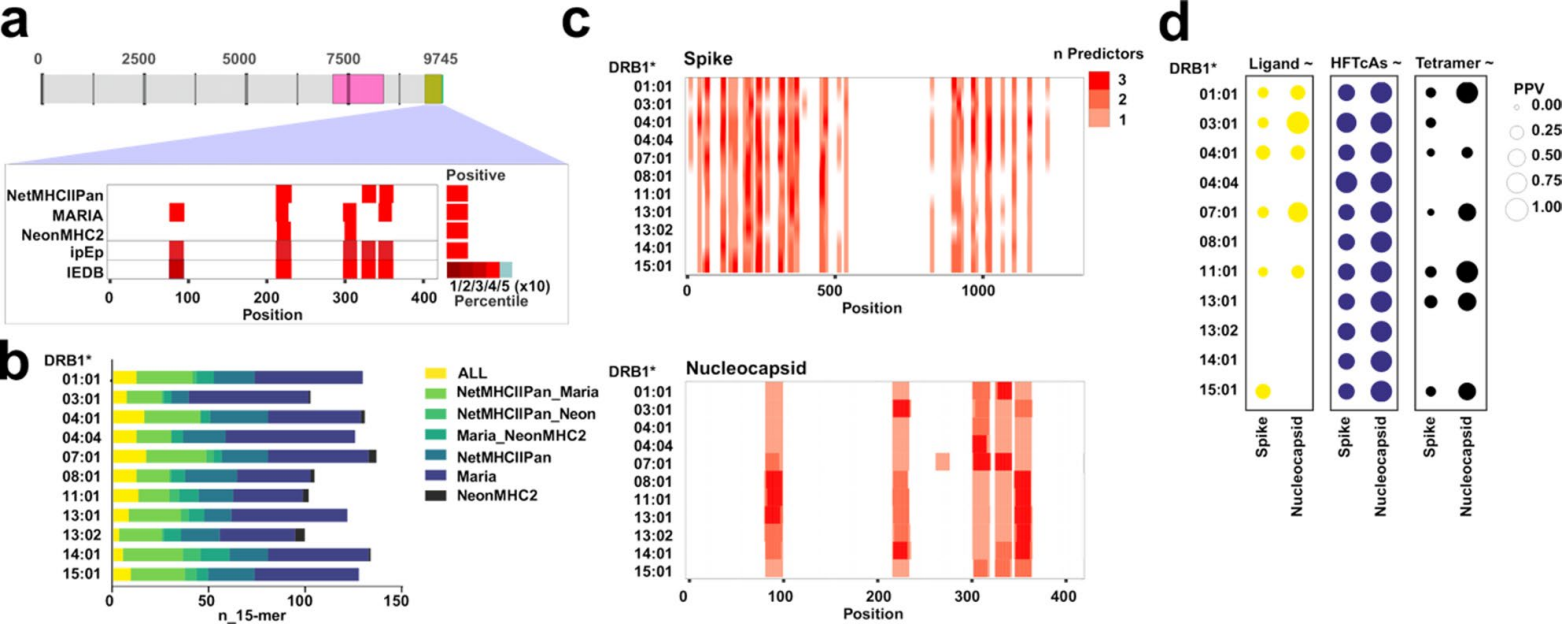
Overview of the in silico predictions.** a**. All potential 15-mers (sliding window of 1 amino acid) of the model antigens (shown in color and in the context of the SARS-CoV-2) were queried in silico as MHCII-binders/presented peptides using three different tools (NetMHCIIPan, MARIA and NeonMHCII). The IEDB combined score of the CD4^+^ T cell immunogenicity prediction tool was used to flag potential candidates. Candidates identified by each of these tools (exemplified by DRB1*03:01 using the nucleocapsid protein), are mapped to each antigen (highlighted in red for the example). The IEDB immunogenicity prediction tool combined score ranges between 0 and 100, and candidates below the 50% percentile are very unlikely immunogenic (see legend). Candidates identified by any of these tools are labelled as in silico predicted Epitopes (ipEp). **b.** Overlap between the different predictors used for each allotype for the Spike and Nucleocapsid proteins shown as a bar-chart. **c.** Correlation between predictors for each DR-allotype, indicated as number of predictors identifying each residue (overlap) for each allotype for the Spike (upper panel) and Nucleocapsid (lower panel) proteins. **d**. Positive Predictive Value of the logistic regression models generated based on predictions to identify residues as: Ligands for individual allotypes where there is available information (Lig); Prevalent T cell epitopes (HF_TcAs, identified in pools of more than 10 individuals as eliciting responses in more than 5); Tetramers including peptide and MCHII allotype information (Tet_TcAs)

We considered using in silico prediction tools as a first line of evidence to gain insights on immunodominance patterns of human responses to the Nucleocapsid and Spike proteins of SARS-CoV-2. These tools have a great potential to identify binding motifs and recent efforts were made to train them on peptides presented at the cellular level. Thus, we tested the potential to become immunogenic of all 15-mers (average size of MHCII-epitopes) from the model antigens and for every DRB1 allotype in our panel using MARIA [10], NeonMHCII [11] and NetMHCIIPan4.0 [12]. Any positive hit for either tool was mapped and considered as a potential in silico predicted Epitope (ipEp; e.g., Fig. 2a, upper panel- Nucleocapsid as model antigen for DRB1*03:01). Despite their individual utility, these tools showed surprisingly poor concordance (lower than 5% for some llotypes between MARIA and NeonMHCII) in identifying overlapping antigenic regions (Fig. 2b). Since all ipEp were flagged by the IEDB immunogenicity prediction tool (based on supertype motifs and sequence similarity searches for known epitopes) we considered that at first sight there is no clear advantage on using any particular tool. Poor overlapping performance was validated across the entire SARS-CoV-2 proteome suggesting that there is no antigen-specific bias (Additional File 1: Fig S2a).

The combined output of these tools selects recurrently around 5 (average 22 peptides) and 25 (100 peptides) regions (consecutive and non-interrupted appearance of 15-mers, with different degrees of overlap between predictors) where ipEp epitopes accumulate for the Nucleocapsid and Spike protein, respectively (Fig. 2c) for our DRB1-panel. A similar picture is observed for the viral proteome with 152 regions and 623 peptides selected by the 11 allotypes of our panel (Additional File 1: Figure S2a). Most ipEp are selected only by one or two of the predictors applied for the model antigens (Fig. 2c) and the entire viral proteome (Additional File 1: Figure S2b). Thus far, MARIA and NeonMHCII show the lowest congruence level with each other (in the range of 2–5%), dropping dramatically for DRB1*13:02 to less than 1% (Additional File 1: Figure S2c). The large amount of information available at the IEDB for those antigens (Additional File 1: Table S2 and S3), enabled us estimating the predictive potential of ipEp to classify IEDB curated entries applying binary logistic regression models. The following type of entries were considered as dependent variables: (i) Ligands for each allotype (subset of those with measured affinities and including only as positive hits those with binding affinities lower than 1000 nM), Lig, (ii) peptides yielding frequent/prevalent responses, HF_TcAs, and (iii) those confirmed via multimer staining, Tet_TcAs. The Positive Predictive Values (PPV) using these models that consider ipEp as independent variable indicate a decent performance of the combination of these tools to score immunogenic peptides (Fig. 2d). There is no clear advantage on using any of them over the rest (Additional File 1: Figure S2d; e.g. for the entire proteome).

### A reconstituted antigen processing system points out different pathways for peptide selection from the Spike and Nucleocapsid proteins

**Fig. 3:**
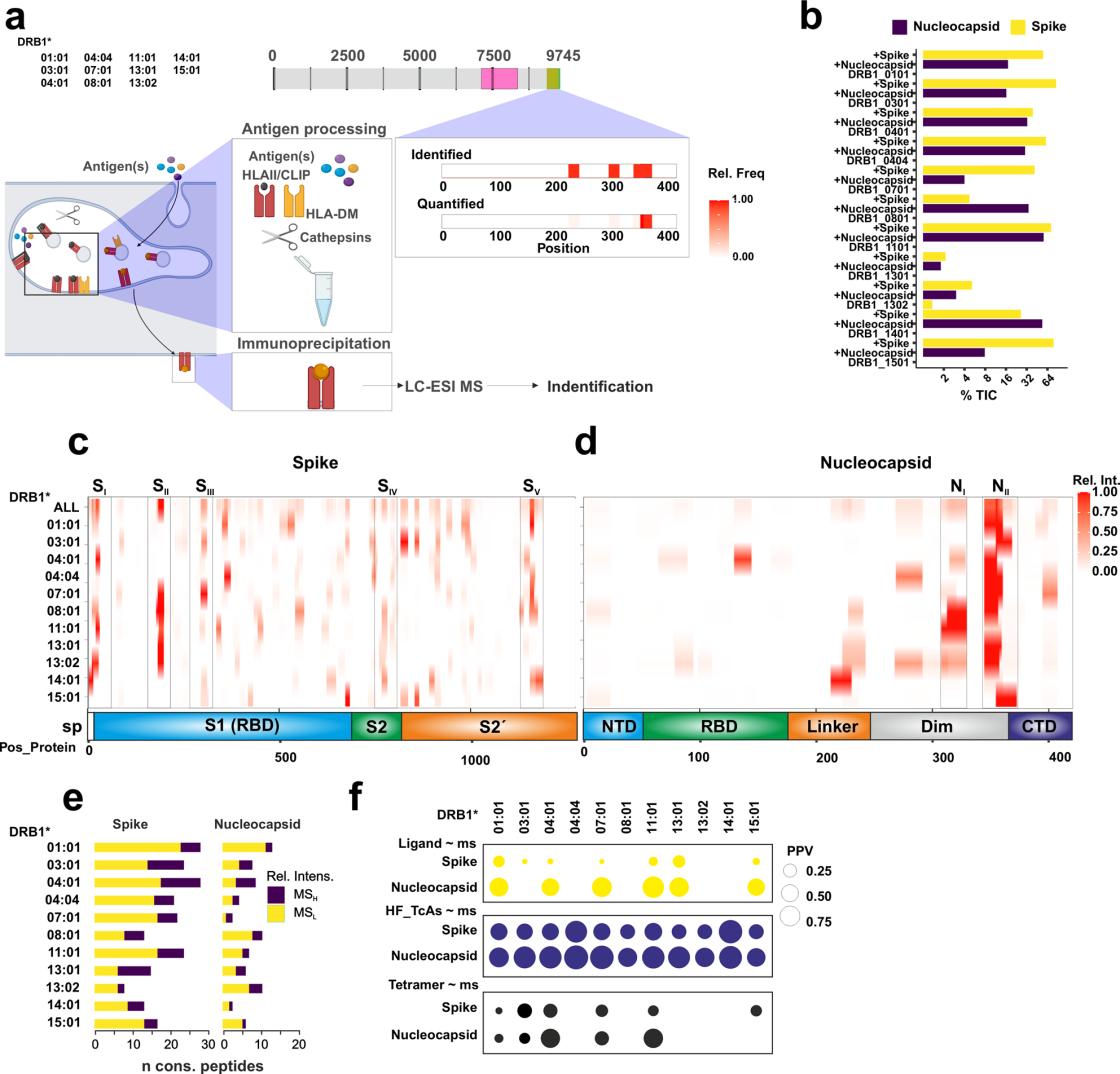
Overview of the reconstituted in vitro antigen processing system. **a**. The protein database used for the searches consists of 311 entries including common MS-contaminants and abundant proteins from the host used for recombinant protein expression (expression host: *S. frugiperda*), those used for protein manipulation and antigen processing proteins as well as all reference SARS-CoV-2 proteins. **b.** Performance of the antigen processing system for the selection of candidate antigenic peptides. The sum of the MS1 intensities for the SARS-CoV-2 peptides is represented for each of the 3 sets of experimental conditions tested. *n* = 2 experiments with 3 technical replicates measured. **c**. Antigenic regions selected by each allotype (left) for the Spike protein and depicted according to their MS1 relative intensity. The lower scheme depicts the main domains of the protein. The sum of intensities of all candidates selected by the 11 allotypes of our panel is shown in upper row, referred as ALL. **d** The same as in c. but for the Nucleocapsid protein. **e** Summary of the number of consensus peptides (maximum of the overlap of the series of nested peptides) identified by MS. The color code refers to the number of peptides defined as those with higher MS1 intensity than the mean (High, MS_H_), or lower (Low, MS_L_). **f** Summary of the Positive Predictive Value (PPV) for the identification of known ligands (Binding affinity below 1000 nM, shown in the upper panel, yellow), epitopes that give rise to prevalent responses (middle panel, blue, HF_TcAs), and those validated by tetramer stains (lower panel, black), based on MS-data for the two antigens used (noted as Ligand⁓ms, HF_TcAs⁓ms, and Tetramer⁓ms). Independent models were attained and tested for each DRB1allotype. The size of the dot refers to the PPV as shown in the legend

We applied a reconstituted antigen processing system previously described for single antigens [17] and tuned by us for identifying T cell epitopes from complex antigenic mixtures [18] to probe immunodominance patterns of human responses to the model antigens using our DRB1-panel. The original system incubates recombinant MHCII molecules with the antigen under study in the presence of the peptide-loading catalyst, DM. Subsequent addition of cathepsins, as main proteolytic functions related to antigen processing select peptides with high likelihood to become immunogenic. This experimental model system does not entirely reflect the cellular environment in its complexity and clearly simplifies the diversity of antigen processing conditions found in Antigen Presenting Cells (APCs). However, it features the main steps of the MHCII-antigen processing and presentation pathway in Dendritic Cells, model APCs with a prominent role on viral defense. The original system enables the interplay of flexible and accessible antigenic regions with the binding groove of MHCII molecules since those are presumably “empty”. Our model follows the same scheme but considers MHCII molecules pre-loaded with the place-holder peptide CLIP resembling more accurately an endosomal-like environment (Fig. 3a). Thus, we followed previously described protocols facilitating the interaction of the antigen with CLIP-loaded MHCII molecules in the presence of DM prior to adding proteases [18]. After immunoprecipitation of pMHCII complexes, peptides are eluted from MHCIIs, cleaned up and evaluated via liquid chromatography coupled to electro-spray ionization mass spectrometry (LC-ESI-MS).

We detect a significant exchange of CLIP for peptides derived from the SARS-CoV-2 antigens applied. On average the total ion current (TIC) for SARS-CoV-2-derived peptides from these antigens reached more than 50% with only two deviations from this behavior (DRB1*13:01 and DRB1*13:02) with TICs for non-CLIP peptides lower than 10% for both antigens (Fig. 3b). This low exchange rate of CLIP for these allotypes was observed with two batches of the corresponding proteins assayed in triplicates validating the outcome of the experiments (see summary in Additional File 1: Table S4). Our analysis pipeline combines the information on the series of nested peptides identified by MS for each allotype into consensus peptides as previously described [29]. Consensus peptides, also referred to as experimentally predicted epitopes (epEp), were then mapped to their corresponding antigen, Spike (Fig. 3c) or Nucleocapsid protein (Fig. 3d) along with their relative intensity for each allotype. Additionally, we combined all relative intensity values for each residue for all allotypes assayed (MS_ALL). This analysis revealed five regions of more than 15 amino acids from the Spike protein (S_I_ to S_v_) that are preferentially selected by more than 4 different allotypes (relative intensity above the median of the candidates for each allotype). Additionally, we observe a clear bias towards the selection of peptides from two regions of the Nucleocapsid protein (N_I_ and N_II_) (details for these regions in Additional File 1: Figure S3). Interestingly, the N_II_ region, selected by all DRB1 allotypes, map to a specific segment of the dimerization domain. Thus, while we detect an allotype-specific pattern of peptide selection for the Spike there is a clear bias to a defined region (N_II_) in case of the Nucleocapsid protein (Fig. 3c and d).

We hypothesized that peptides preferentially selected by this experimental model represent regions with a higher likelihood to be displayed to T cells in a cellular environment and thus become epitopes (Fig. 3e). To test this hypothesis, we used again binary logistic regression models evaluating the performance of the MS-intensity values to inform about residues that have been described as Ligands (Lig), entries yielding frequent/prevalent responses (HF_TcAs) and via multimer staining, Tet_TcAs. Together, epEp defined by this model using MS reach PPV that go up to 0.75 to identify residues from peptides reported as HF_TcAs (Fig. 3f). Thus, we conclude that the reconstituted antigen processing system selects peptides resembling those of HF_TcAs albeit no available information exists for them as binders or Tetramer. However, since these peptides are eluted directly from the MHCII molecules tested we interpreted them as ligands with the potential to be immunogenic restricted by the corresponding MHCII allotypes.

### The interplay between in silico and in vitro predicted epitopes delineates a minimalistic peptide pool with broad population coverage

**Fig. 4:**
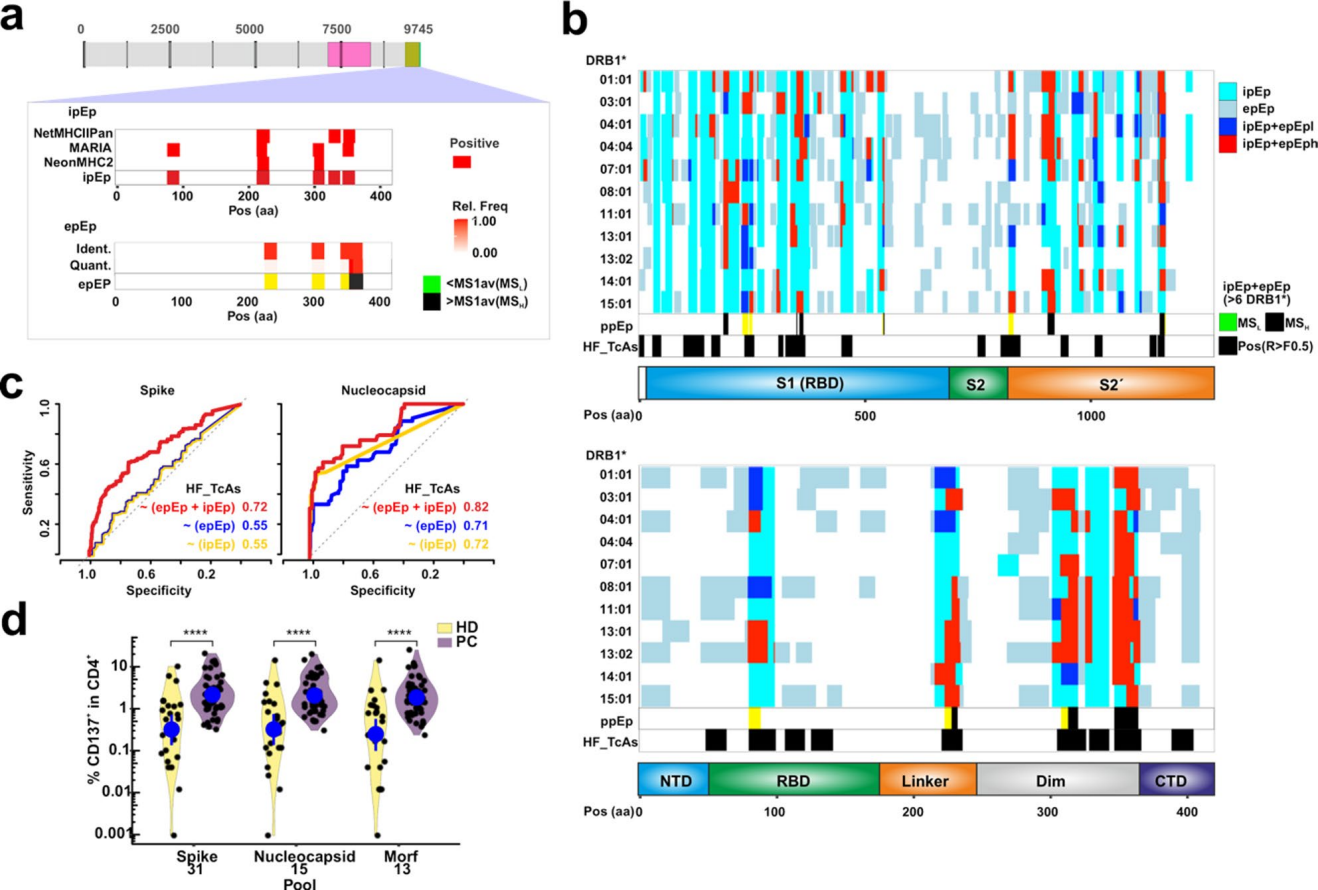
In silico and experimental strategies to define potential Epitopes (pEp).** a**. Strategy followed for the identification of potential Epitopes (pEp) by computational and experimental methods. Three in silico predictors (NetMHCIIPan, Maria and NeonMHCII) are used to identify 15-mers (sliding window of 1 amino acid) from the Spike (pink) and Nucleocapsid (orange) proteins with immunogenic potential. Candidates identified by any of these tools are considered in silico predicted epitopes (ipEp). Experimentally, we applied a reconstituted antigen processing system yielding qualitative and quantitative information and defining regions preferentially selected for presentation, hence experimentally predicted epitopes (epEp). For epEp, if MS1 of a region is higher than the average of the peptides identified by MS then we considered this region as high intensity MS_H_, and if it was lower MS_L_. The example shows ipEp and epEp for DRB1*03:01 using the Nucleocapsid protein with each of the in silico tools and the outcome of the reconstituted antigen processing system. **b**. Overlap between predictions and experimental data highlights regions of promiscuous predicted Epitopes (ppEp) and existing data on recurrent responses previously measured (HF_TcAs) with a response frequency higher than 50% and tested in at least 10 individuals. Note that we considered here as well MS_H_ and MS_L_ as indicated in a). **c** ROC curves depicting the performance (as AUC) of ipEp, epEp and their combination to define HF_TcAs from each of the antigens considered based on Binary logistic regression models. See legend for AUC values. **d** Frequency of activated (CD137^+^) CD4^+^ T cells upon stimulation with each of the pools indicated on the x-axis as determined by flow cytometry. Two groups were tested for each peptide pool, individuals non-previously infected with SARS-CoV-2 (HD, Healthy donors, yellow, *n* = 24) and individuals that had recovered from SARS-Cov-2 infection (P.C., Post-Covid19, purple, *n* = 48). The difference between the median of the responses was compared applying a non-parametric Mann-Whitney test, and the significance is reported as follows: *p < 0.05; **p < 0.01; ***p < 0.001; ****p < or = 0.0001. Values for individuals with no response detected where set to 0.001

We examined the interplay between ipEp and epEp to define optimized peptide pools with broad population coverage. Using the Nucleocapsid protein and DRB1*03:01 as a representative example, we observed partial but consistent overlap between the predicted epitope subsets (Fig. 4a), suggesting complementary predictive performance. When we considered all allotypes tested, this strategy highlights regions recurrently selected by several DRB1 molecules, which aligned well with previously defined HF_TcAs (Fig. 4b) suggesting that the combined predictive framework reliably captures CD4^+^ T cell epitopes. Provided the MS1 intensities as measured in the reconstituted in vitro system, we defined MS_H_ and MS_L_ (referring to high and low abundance selected candidates). Using logistic regression models, we estimated the probability of a residue being part of an immunodominant region (HF_TcAs or Tet_TcAs) when considering ipEp, epEp or their combination. The improved performance observed — quantified by AUC-ROC values — for the combination of both predictions as compared to either method alone validates our strategy (Fig. 4c). However, the limited availability of Tet_TcAs restricted robust evaluation of allotype-specific predictions even though a similar scenario would be expected according to the PPV observed for those when using epEP or ipEP.

Given the benefit of the combination of ipEp and epEp to point out residues contained in immunodominant epitopes, we used their overlap to design a minimalistic peptide pool (MPpN for the Nucleoprotein and MPpS for the Spike consisting of 15 and 31 peptides) with broad phenotypic coverage. We allowed flexible peptide sizes (between 15 and 20 residues) to accommodate diverse HLA restrictions. A subset of peptides from relatively small proteins of SARS-CoV-2, determined exclusively in silico (as ipEp), was included as control (M, and orf3a and orf7 proteins consisting of 14 peptides, Additional File 1: Figure S5 and Table S5). Flow cytometry activation assays performed on samples retrieved from our panel of donors revealed a recurrent ability of the three pools to trigger T cell responses in PC individuals compared to HD (Fig. 4d). Activated CD4^+^ T cells from PC ranged between 2 and 15%, reaching similar frequencies of responders as previously shown by others [21]. Increased levels of all cytokines, activation and exhaustion markers tested for all PC individuals when compared to those of HD, validate our minimalistic peptide pools conceived for our broad coverage DRB1-panel (Additional File 1: Figure S6).

### Validation of immunodominance patterns reveals alternative mechanistic models of peptide selection

We validated the immunodominant potential of our MPpN and MPpS candidates to test the performance of our platform, aiming to gain insights into their potential selection mechanisms. To minimize the impact of factors that may bias immune responses (e.g., binding competition), we selected individuals sharing the highest number of DRB1 allotypes, namely DRB1*03:01-*07:01. This combination is present in 2 HD and 3 PC with only partial divergences on HLA-DP (Additional File 1: Figure S7a).

**Fig. 5:**
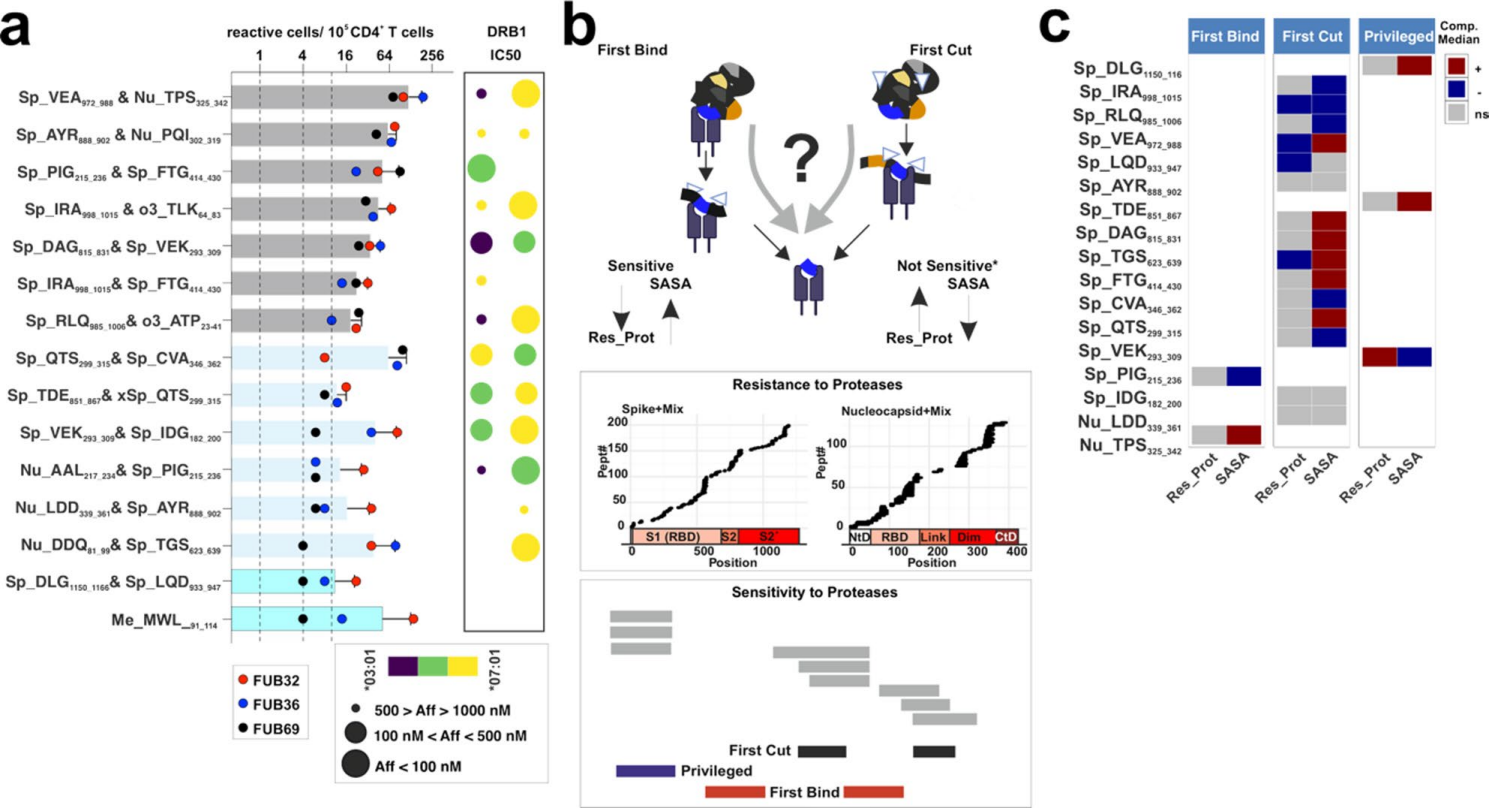
Immunodominance and molecular insights on the selection of allotype-specific peptide pools.** a** Frequency of responder cells to each dual peptide combination measured via ELISpot (IFNg + IL-10) from PC donors. The number of spots is converted to responder cells per million of PBMCs and the frequencies determined for every individual (measured in duplicates) are shown according to the color code depicted in the legend. Dashed lines represent thresholds allowing the classification of the measured response. The first line at “10” is the maximum of responder cells detected in any negative control (HD), the second line is the minimum observed response for any combination, and the third line represents a 2-fold increase of Line 2. The dual peptide combinations are indicated on the left side and the color code of the bars indicate the distinct immunodominant responses measured (Dark gray and those immunogenic (light gray). Potential restrictions defined by IC50 determination over the two DRB1* allotypes present in these donors. Each dot represents the affinity of each peptide for either allotype as depicted by the size and color (see legend), Note that affinity differences lower than 1.5-fold are considered as possibly restricted by both allotypes (shown in green). **b** Antigen-intrinsic and -processing related features defining mechanistic models for peptide selection depicted as scheme. Proteolytic digestion of the two antigens tested reveals peptides resistant to proteases under the tested conditions (Res_Prot) and regions sensitive to proteases that point out at the different mechanistic models. Residues found through more than 3 peptides within series of nested peptides longer than 7 residues are considered indicative of the “First Cut” model. Disruption of series of nested peptides in more than 3 peptides are indicative of the “First Bind” model. Remaining regions with represented in more than 3 peptides with a full coverage of an antigen are considered “Privileged”. **c** Antigen processing mechanism and antigen-intrinsic features of every peptide tested in the dual combinations from the two model antigens. Antigen sources for every peptide are indicated as: Nu-, Sp-, o3- and Me- for Nucleocapsid, Spike, orf3a and Membrane, respectively. The first three residues of the peptide and the positions are also indicated. Res_Prot and SASA values for each candidate are compared to those of a random selection of peptides excluding all known epitopes (IEDB accession Sept. 2022) and represented according to the scale shown in the right of the panel. “+” indicates higher than median, “-“ refers to values lower than the median and “ns” stands for not significant (significance tested through a Wilcoxon Rank test)

Allotype-specific pools feature high and recurrent responses in samples from PC individuals compared to those of HD, as measured via dual-color ELISpot (Additional File 1: Figure S7b-c). The same observations were made using dual peptide combinations. Dual peptide combinations triggering two-fold response frequencies higher than the controls for all PC individuals were considered immunodominant. Two combinations clearly deviate from this criterion (boxed bars, Fig. 5a). We defined two subsets of immunodominant peptides: (i) those with frequencies of responder cells four-fold higher than any control for all PC tested individuals (7 combinations, 13 peptides), and (ii) those with divergent profiles for every individual (gray and light blue bars respectively, Fig. 5a). Most combinations bear at least one peptide with high or medium binding capacity towards either DRB1*03:01 or *07:01 as determined by IC50 measurements (18 peptides out of 23, IC50 < 1000nM, Additional File 1: Figure S7d). We assume that peptides with no IC50 value for blocking reporter peptide binding may be presented by any of the additional allotypes from the PC individuals (limited to HLA-DP molecules). Altogether, despite the potential bias in T cell clonotype frequencies in these individuals, the observed patterns validate the postulated pipeline and immunodominance of most of the selected candidates.

Next, we considered endo-protease cleavage (Sensitivity) and resistance to proteases (Res_Prot, experimentally determined by us, details in Supporting Information and in Additional File 1: Figure S8) as well as solvent accessible surface accessibility (SASA) as representative parameters to define the molecular mechanism favoring antigenic peptide selection (Fig. 5b). We first evaluated the antigen degradation profiles to determine regions that do require to be protected from proteolysis, hence selected most likely through the FBtc model. Then, we delimited those that arise from regions partly resisting proteolysis under the tested conditions and classify them as primarily selected by the FCtb model. Surprisingly, more than 2/3 of the candidates confer to the second mechanism (Fig. 5c). A third subset of peptides is identified that could be considered selected via either of the two models since they are capable of withstanding proteolysis under the assayed conditions, and thus we termed them “Privileged”. Altogether, we validate that our MPp have a higher proportion of peptides selected through the FCtb model.

### CD4^+^ immunodominant epitopes are selected through different pathways for the nucleocapsid and Spike protein

Our results indicate that the selection of antigenic peptides triggering immunodominant CD4^+^ T cell responses occur primarily through the so called FCtb model, consistent with a previous work focused on Influenza´s Hemagglutinin with DRB1*01:01 [9]. However, the importance of FBtc mechanism has been recently thoroughly demonstrated [8, 13]. Besides, to the best of our knowledge there was no prior report on the so-called “Privileged” peptides in humans. Under these premises we determined whether and to what extent these observations hold at the human population level for these antigens.

**Fig. 6:**
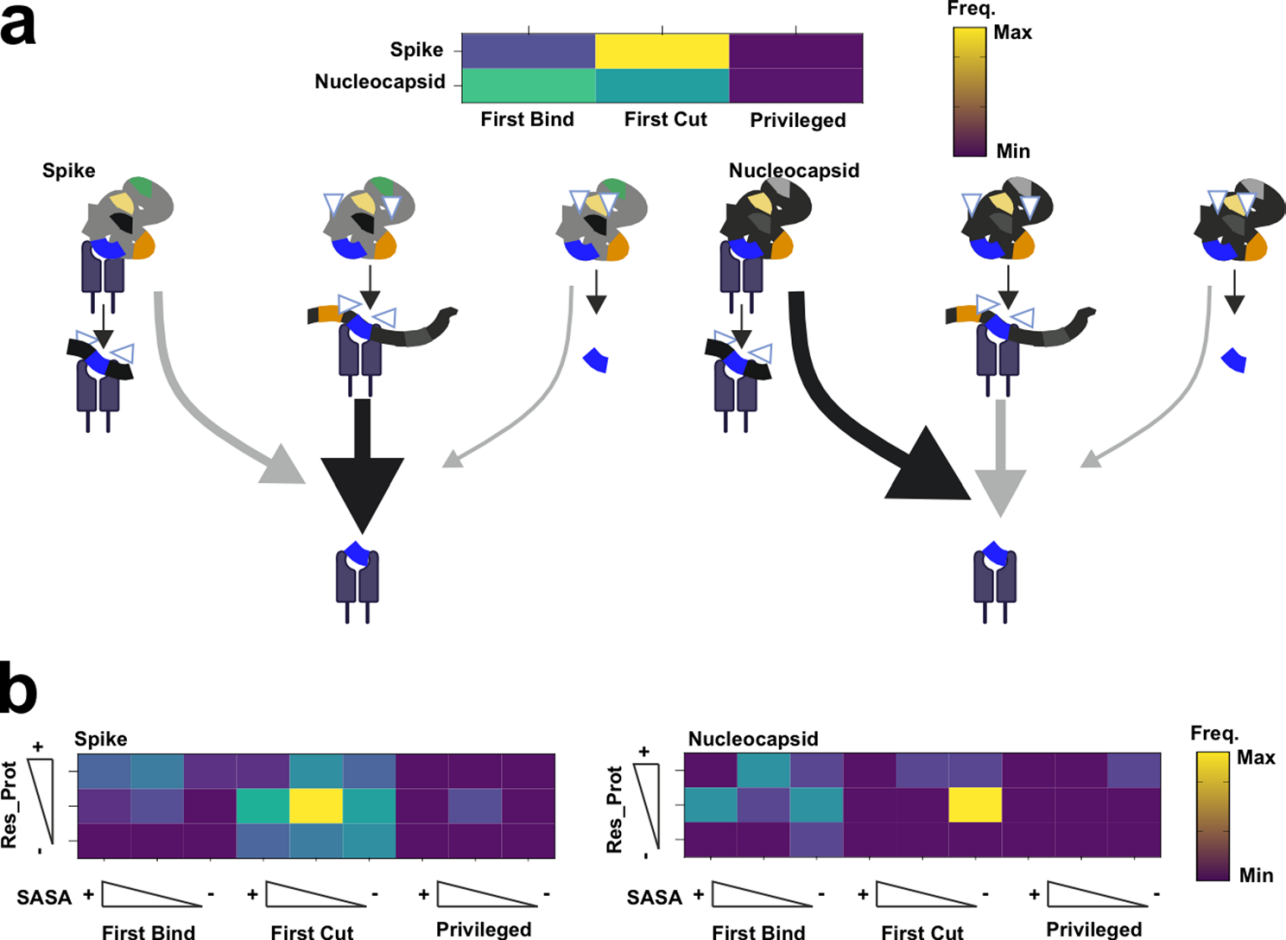
Evaluation of the different mechanistic models through all Spike and Nucleocapsid reported epitopes. **a**. Frequency of peptide selection model for all epitopes previously described at the IEDB (September 2022) for the Nucleocapsid and Spike protein. Each epitope entry for the Nucleocapsid or Spike protein (HF_TcAs or Tet_TcAs) is mapped to the corresponding antigen to define whether they lie at regions sensitive to proteases, as indication of the peptide selection model (“First Bind”, or “First Cut” as well as “Privileged” peptides). Graphical representation of the main findings on the frequency of the peptide selection mechanism for immunodominant epitopes for each antigen. Black arrows represent the most prominent pathway for each antigen. **b**. Heatmaps as in a) depicting the additional impact of SASA and Res_Prot on the entries selected via each molecular mechanism. For each epitope, averaged values for those features were compared to a random distribution of peptides excluding any epitope resulting in three categories. Those categories include higher “+”, no difference “ “ (middle squares), or lower “-“, significance was tested through Wilcoxon-ranked test

We classified every known HF_TcAs and Tet_TcAs according to the parameter Sensitivity to proteases to validate whether the selection pattern observed for the MPp peptides apply (Additional File 1: Figure S9). Surprisingly, we detect a different distribution on the frequency of the models for each antigen. The FCtb model is clearly prominent path for the Spike protein (83% of the peptides) whereas the FBtc is the preferred one for the Nucleocapsid (55%) (Fig. 6a). Additionally, we observed a very limited frequency of the so-called “Privileged” epitopes (3–4% for both antigens). Thus, we conclude that the preferred processing pathway for the selection of immunodominant peptides depends on the antigen (Fig. 6a lower panel).

Finally, we evaluated whether any of the additional features considered — SASA and Res_Prot — may inform specific insights for the selection of peptides by each mechanism for each antigen. We accounted for the differences on these features compared to the control peptide pool to conclude that the only apparent difference is that peptides selected through the FCtb mechanism are selected from low SASA regions for the Nucleocapsid protein while the selection from the Spike protein does not follow such pattern (Fig. 6b). In conclusion, these results derived from hundreds of human responses indicate that antigen-specific features impose distinct selection patterns. It is obvious that the limited number of antigens tested default broader generalizable statements on antigenic features favoring these pathways. The higher Res_Prot and SASA observed for the Nucleocapsid protein compared to the Spike should be responsible for the preferential selection through the FBtC mechanism (Additional File 1: Figure S10). Prospective studies should aim at the systematic evaluation of the influence of these parameters on the selection mechanism of immunodominant epitopes from different antigenic sources.

## Discussion

Our work establishes and validates a robust pipeline for the mechanistic analysis of human CD4^+^ T cell immunodominance. By integrating experimental and in silico information across a panel of MHCII allotypes with diverse binding specificities and high population coverage, we provide new insights into the selection and presentation of immunodominant epitopes using the Spike and Nucleocapsid proteins of SARS-CoV-2 as model antigens. We demonstrate that while in silico predictions remain limited by algorithm-specific biases, our experimental workflow effectively narrows down antigenic regions of interest, even across MHCII allotypes with divergent binding grooves. The combination of the two approaches enables the rational design of a minimalistic peptide pool featuring a remarkable population-wide immunodominance profile. Contextualizing antigen and antigen-processing features of peptide candidates confirms that CD4^+^ T cell immunodominance is not restricted to a single molecular mechanism, and that that the specific mechanism depends on the antigen considered. Altogether, we conclude that alternative pathways for epitope selection play a key role and should be considered for improving immunodominant epitope discovery.

Identifying immunodominant CD4^+^ T cell patterns at the human population level has important implications for basic and applied research. Allotype-specific immunodominance has limited applicability at the population level unless broad panels of MHCII allotypes are considered. Haplotype immunodominance on the other hand lacks insights on restrictions offering limited applicability for personalized interventions. We integrate both views of immunodominance not only to identify peptide selection patterns, but also to score mechanistic insights [8, 9, 13] at a population-wide level. Our choice of antigens was mainly motivated by the availability of data, with more than 1800 CD4^+^ epitope entries and approximately double the number of T cell assays (available at the IEDB by September 2022). Besides, our DRB1 panel shows an extraordinary phenotypic coverage throughout our donors, and in the most representative studies published so far (more than 80% in [21]), enabling the comparison of the extent of the responses detected. We capitalize on the fact that SARS-CoV-2 reactive T cells expanded during the first months of the pandemics are expected to result from naïve clonotypes since no previous contact to this pathogen had been reported. However, cross-reactivity with other coronavirus and previous infections [30–35], but also the interplay of the host with its own microbiota [36] may still impose altered backgrounds explaining inter-individual differences of the measured responses.

Our analysis revealed very limited overlap for three state-of-the art tools in silico prediction tools (Fig. 2 and S2), underscoring the lack of consistency across platforms. These discrepancies likely stem from differences in training datasets, algorithmic design, and input parameters — factors that lie beyond the scope of this study (for details see references [10–12]). From an experimental point of view, we incorporated the natural placeholder peptide CLIP [8, 13, 17, 37], with a considerable impact on epitope selection (reviewed in [38, 39]), and managed to reduce the background of self-antigenic peptides, typically representing more than 90% of the identified pepties on immunopeptidome studies [40, 41]. However, we are aware of the limited subset of processing conditions tested in our experiments [5, 42] and that post translational modifications occurring in cells (affecting antigen processing, immunogenicity and immunodominance) may have escaped our approach [43]. Altogether, despite the limitations described for our experimental system, immune response data compiled in the Immune Epitope Database (IEDB) supports its superior performance. Moreover, integrating both in silico and experimental outputs significantly enhances epitope identification, validating the strength of our combined approach.

Prioritizing experimental information and allowing different peptide lengths we achieve a 10-fold reduction of peptide numbers, and more importantly reached similar T cell activation levels as those reported under very similar experimental conditions with larger pools (measured as % of CD137^+^ in CD4^+^ cells in the range of 0.5–10%) [44, 45]. Thus, our strategy clearly optimizes the resources needed for the assessment of antigen immunogenicity, especially for complex antigens (see below). Targeted validation of the immunodominant potential of selected peptides was performed on a very specific MHCII background (DRB1*03:01/DRB1*07:01) to prevent MHCII binding competition. The set of 23 peptides (over 59) consisted of two sub-pools covering both DRB1 restrictions present in the donors. These candidates featured an excellent performance for re-calling CD4^+^ T cell immune responses. Potential restrictions were proposed by measuring the ability of each peptide to block reporter binding to each allotype. 6 peptides out of 23 had neglectable or very low binding affinity for the DRB1 allotypes considered under the assayed conditions but they are capable to activate CD4^+^ T cells (S_933 − 947_, and S_414_430_, S_623_639_, S_1150 − 1166_, N_339 − 361_ and M_91 − 114_). Since most of them had been identified as eluted from these DRB1 molecules in the experimental approach (all except the M_91 − 114_), we conclude that they represent poor-binders and/or intermediate products of antigen processing hot-spots (e.g. preferentially selected upon binding and proteolysis). Together, these results and observations validate the performance of our approach and highlight that there is still room for improvement. Future studies should aim at a broader systematic testing on similar MHCII backgrounds, and scoring the tradeoffs of different peptide lengths for covering broader spectrum of restrictions facilitating clinical applications related to diagnostics or prevention.

Strategies to assess immunodominance of complex pathogens/antigens, where hundreds of proteins are surveyed by the immune system, require additional considerations. These cases may require systematic testing of immunogenicity of virtually all antigens on typed individuals. Most conventional approaches imply the use of overlapping peptides covering relevant antigens. More than 2,000 (14-mer overlap between peptides), or several hundreds of peptides - depending on whether full or partial coverage - are required for the two model antigens tested here [46–49]. In case of complex antigens, as the one studied by us in [18] and consisting of more than 250 proteins, these numbers scale up hundreds or dozens of thousand, limiting the feasibility of these strategies. Evidence exists for immunodominance being influenced by the spatial-temporal expression and abundance of the different antigens [20, 50, 51]. However, even if protein expression, abundance and competition for binding influence peptide presentation, pMHC display does not always correlate with immunodominance [52]. In these cases, likely the preferential targeting by the immune system needs to be accounted for (e.g., cell and mechanism targeting the antigen and clonotype diversity).

Evolutive pressures represents an additional challenge for the assessment of immunodominance patterns of high mutational rate pathogens. During the SARS-CoV-2 pandemics, the tracking of variants of concern (VoC) facilitated scoring the contribution of specific mutations to immune evasion, including CD4^+^ T cell responses. Despite mutations on specific amino acids affecting MHCII restriction or T cel recognition, the broad antigenic coverage at the individual level enables effective immune responses [53, 54]. The presence of certain mutations from VoC in the peptides selected by our scheme (Additional File 1: Table S5), it is likely that those will not entirely compromise individual´s capacity to fight infection as seen in our results. However, individual-specific responses restricted by specific MHCII restrictions may be affected.

Antigen processing constraints play a pivotal role in peptide selection by MHCIIs, thereby facilitating epitope discovery [8, 37, 55, 56]. Two recent studies have demonstrated the impact of antigen processing constraints on the selection of immunodominant epitopes. Cassotta et al. [9] used an Antigen Processing Likelihood [55] estimate to conclude that immunodominant peptides are predominantly located close to, but not directly on, unstable protein regions of Infuenza’s Hemagglutinin. Proteolytic cleavage at endosomal compartments is required for binding implying a mechanism that could be interpreted as FCtb. Sengupta and collaborators [13] on the other hand report that immunodominant peptides from the HIV proteome lie mainly at unstable regions that bind directly to MHCIIs (FBtc). Previously, the same group reported that pathogen derived epitopes would be sensitive to proteases [8]. Our analysis confirms that known epitopes and those that we validate, exhibit features associated with different molecular mechanisms. Indeed, proteolytic degradation – limited conditions – reveal that selection of immunodominant peptides from either antigen are selected through a preferential and specific molecular mechanism. Future works expanding our knowledge on CD4^+^ T cell epitopes [27], combined with tools as AlphaFold enabling efficient structure predictions [57], and improved knowledge on proteolytic functions will equip us with more accurate information.

We demonstrate the potential of integrating experimental and in silico approaches to effectively assess and understand immunodominance in an almost naïve T cell repertoire. Despite challenges for scoring immunodominance at different levels, our findings underscore that the impact of antigen-related features (e.g. SASA) and experimentally definable processing constraints (e.g. proteolytic degradation) should be funneled into the next generation pipelines of effective MHCII-immunodominance prediction. Integrating these factors in predictive models could significantly enhance the precision of epitope identification, ultimately improving our capacity to design vaccines and immunotherapies tailored to individual HLA haplotypes. This perspective may prove particularly valuable for the development of personalized T cell-based responses in the face of future pandemics or in the context of cancer immunotherapy.

## Conclusions

By integrating in silico predictions with a reconstituted antigen processing system across multiple HLA-DRB1 allotypes, we provide a mechanistic framework for understanding CD4⁺ T cell immunodominance in humans. Our findings reveal that distinct antigen-specific processing pathways — namely First Cut then bind or First Bind then cut— govern peptide selection, and that incorporating these mechanistic insights enhances the precision of epitope prediction. This integrative approach supports the development of minimal, broadly immunogenic peptide pools and sets the stage for more effective immunogenomic profiling, immune monitoring, and rational vaccine design across diverse populations.

## Supplementary Information

Additional File 1 includes: Box 1. Glossary of terms used, Figures S1 to S10, and Tables S1 to S5.

## Data Availability

The datasets used and/or analyzed during the current study are available from the corresponding author on reasonable request.

## Abbreviations

AIC: Akaike Information Criterion
AUC: Area Under the Curve
Bind. Motif: Binding Motif
CLIP: Class II Invariant Chain associated Peptide
DMSO: Di-methyl-Sulfoxid
EDTA: Ethylenediaminetetraacetic acid
ELISpot: Enzyme-Linked ImmunoSpot
epEp: experimentally predicted Epitopes
FDR: False Discovery Rate
FITC: Fluorescein Thio-isocyanate label
FP: Fluorescence Polarization
HD: Healthy Donors
HLAII: Human Leukocyte Antigen of class II
HF_TcAs: High Frequency T cell Assay
IEDB: Immune Epitope Database
ipEp: in silico predicted Epitopes
LC-ESI-MS: Liquid Chromatography Electron Spray Ionization Mass Spectrometry
MBP-FITC: Myelin Basic Protein peptide 83–99 labelled with FITC
MHCII: Major Histocompatibility Complex of class II
MOI: Multiplicity of Infection
MPpN: Minimalistic Peptide pool Nucleoprotein
MPpS: Minimalistic Peptide pool Spike
MS: Mass Spectrometry
MWCO: Molecular weight cutoff
PBMC: Peripheral Blood Mononuclear Cells
PC: Post-Covid19
pEP: predicted Epitopes
pMHCII: peptide-MHCII complex
RF: Response Frequency
Rel_Prot_Res: relative Resistance to Proteases
Rel_SASA: relative Solvent Accessible Surface Area
ROC: Reiceiver Operator Curves
SDS-PAGE: Sodium Dodecyl Sulfate-Polyacrylamide Gel Electrophoresis
TcAs: T cell Assays
Tet_TcAs: Tetramer-identified T cell Assay
VoC: Variants of Concern
