# Supplementary material for: Cut or bind? Antigen-specific processing mechanisms define CD4+ T cell immunodominant epitopes for SARS-CoV-2 S and N proteins
Source: Genome Med. 2025 Nov 26;17:147. doi: 10.1186/s13073-025-01577-8 (PMC12676865; doi:10.1186/s13073-025-01577-8)
Supplement: Supplementary file 1 — Additional File 1 includes: Box 1. Glossary of terms used, Figures S1 to S10, and Tables S1 to S5. [file 13073_2025_1577_MOESM1_ESM.docx]

**Cut or Bind? Antigen-specific processing mechanisms define CD4^+^ T cell immunodominant epitopes for SARS-CoV-2 S and N proteins**

Miguel Álvaro-Benito^1,2,3,†,*^, Esam T Abualrous^3,4,5,†^, Holger Lingel^6^, Stefan Meltendorf^6^, Jakob Holzapfel^3^, Paula de Diego Valera^1,2,3^, Jana Sticht^3^, Benno Kuropka^7^, Cecilia Clementi^8^, Frank Kuppler^3^, Monika C Brunner-Weinzierl^6^, Christian Freund^3^

^1^Department of Immunology, Ophthalmology and ENT, Universidad Complutense de Madrid School of Medicine

^2^Lymphocyte Immunobiology 12 de Octubre Health Research Institute (imas12); Madrid, Spain.

^3^Laboratory of Protein Biochemistry. Department of Biology, Chemistry and Pharmacy. Freie Universität Berlin; Berlin, Germany.

^4^Department of Mathematics and Computer Science, Freie Universität Berlin; Berlin, Germany.

^5^Department of Physics, Faculty of Science, Ain Shams University; Cairo, Egypt.

^6^Department of Experimental Pediatrics, Medical Faculty, Otto-von-Gericke-University; Magdeburg, Germany.

^7^Mass Spectrometry Core Facility (BioSupraMol), Freie Universität Berlin; Berlin, Germany.

^8^Theoretical & Computational Biophysics, Institute for Physics, Freie Universität Berlin; Berlin, Germany.

Corresponding author: Miguel Álvaro-Benito. c/ Severo Ochoa s/n, Department of Immunology, Ophthalmology and ENT, Universidad Complutense de Madrid. 28040, Madrid. [migalv07@ucm.es](mailto:migalv07@ucm.es)

**This PDF file includes:**

Box 1. Glossary of terms used in this manuscript

Figures S1 to S10

Tables S1 to S5

References

**Box 1. Glossary of terms used in this manuscript**

Our manuscript eludes on biochemical aspects, specific terms and concepts of the field of antigen processing and presentation. To ease the understanding and readability for immunologists not related to the field we include the following glossary of concepts and terms.

**Working hypothesis:** Antigenic peptide selection by MHCII molecules (pMHCII) triggering dominant immune responses is influenced by processing mechanisms that depend on the structural and biophysical features of the processed target protein. Knowledge about these features can be derived by a reconstituted experimental system. When combined with binding data, optimized antigen pools for raising T cell responses can be obtained.

**CD4^+^ T cell Immunodominance:** Preferential and recurrent T cell response to specific antigenic determinants. Immunodominance results from the specific selection of antigenic peptides defined by the presence of MHCII allotypes and antigen processing, as well as the presence and engagement of cognate T cell receptors of those pMHCII. Note that immune responses against pathogens are often modulated by the presence of clonotypes raised against similar antigens (molecular mimicry), T cell receptor cross reactivity and even the lack or preferential selection of certain clonotypes in T cell selection.

**T cell epitope:** Is the molecular entity recognized by a T cell Receptor and triggering T cell activation. T cell epitopes, consists of the combination of a given MHCII molecule bound to a peptide.

**Phenotypic coverage:** proportion of individuals having at least one copy of an allelic variant of a gene. When considering a panel of allotypes the term refers to the proportion of individuals bearing at least one copy of those.

**MHCII supertypes**: groups of molecules with overlapping binding specificities that define averaged binding motifs.

**Molecular mechanism of peptide selection:** Biochemical and structural processes, including protease sensitivity and binding interactions, that determine which peptides are processed and presented by MHCII molecules to the immune system. Early work using model antigens and animal models revealed that peptides within an antigen are selected depending on their position and the nature of adjacent regions.

**First Bind then cut (FBtc):** antigenic peptides can bind to MHCII molecules at either the cell surface or within endosomal compartments if they are sufficiently exposed to the solvent or in flexible regions. This binding protects the antigens from being degraded while the rest of the antigen is cleaved by endo- and exo-proteases.

**First Cut then Bind (FCtb):** antigenic peptides within buried or un-exposed regions cannot bind directly to MHCII molecules. Therefore, cleavage of adjacent regions leading to unfolding of the protein or exposure of the corresponding peptide facilitates binding to MHCII molecules.

**
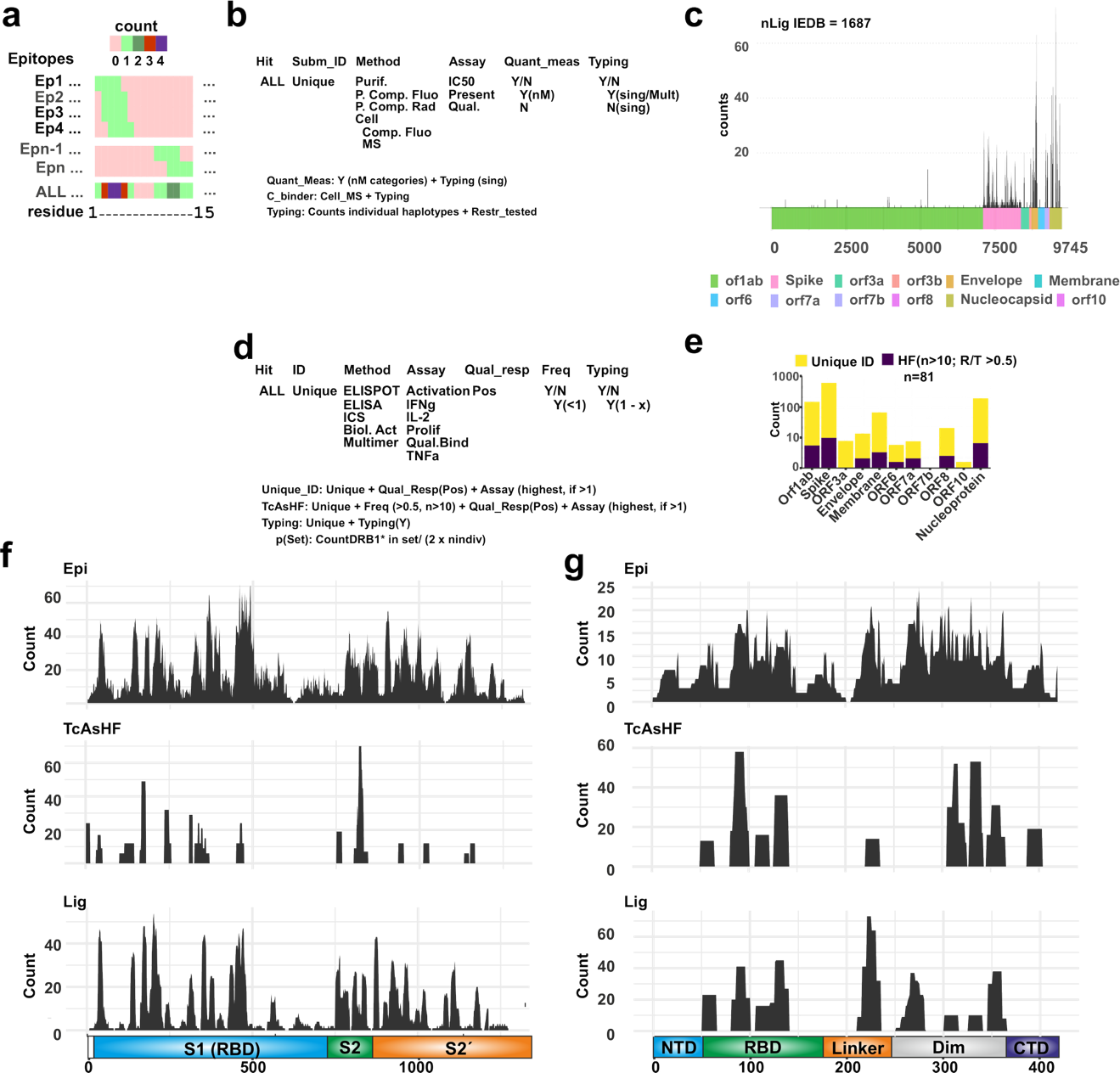
**

**Fig S1. Summary of the retrieval of IEDB data for its use**. **a**. Vector coding scheme of all available entries. **b.** Scheme of the data arrangement downloaded and its usage for downstream applications in case of Ligands. There are three subsets of information available and the filtering criteria for each of them is considered. **c**. Schematic representation of the counts of measurements for each residue within the ligand report. **d**. Same as in b) but in this case referred to data derived from T cell assays (TcAs). **e**. Counts of T cell Assay data for each orf considered where the height of the bar represents the total number of unique hits. Those considered as high frequent hits are shown in purple (HF, n individuals tested higher than 10 and at least 5 of them with a positive response). **f.** Zoom in the IEDB counts for each of the entries considered for the Spike protein. Epi: epitope, TcAsHF: High Frequency T cell Assays, and Lig, ligands. The protein is shown in the lower part of the panel with the main domains represented (S1 or RBD- Receptor Binding Domain), S2 and S2´. **g.** As f but for the Nucleocapsid Protein. In this case the domains depicted are NTD: N-terminal Domain, RBD: Receptor Binding Domain, Linker, Dim: Dimerization motif, and CTD: C terminal Domain.

**
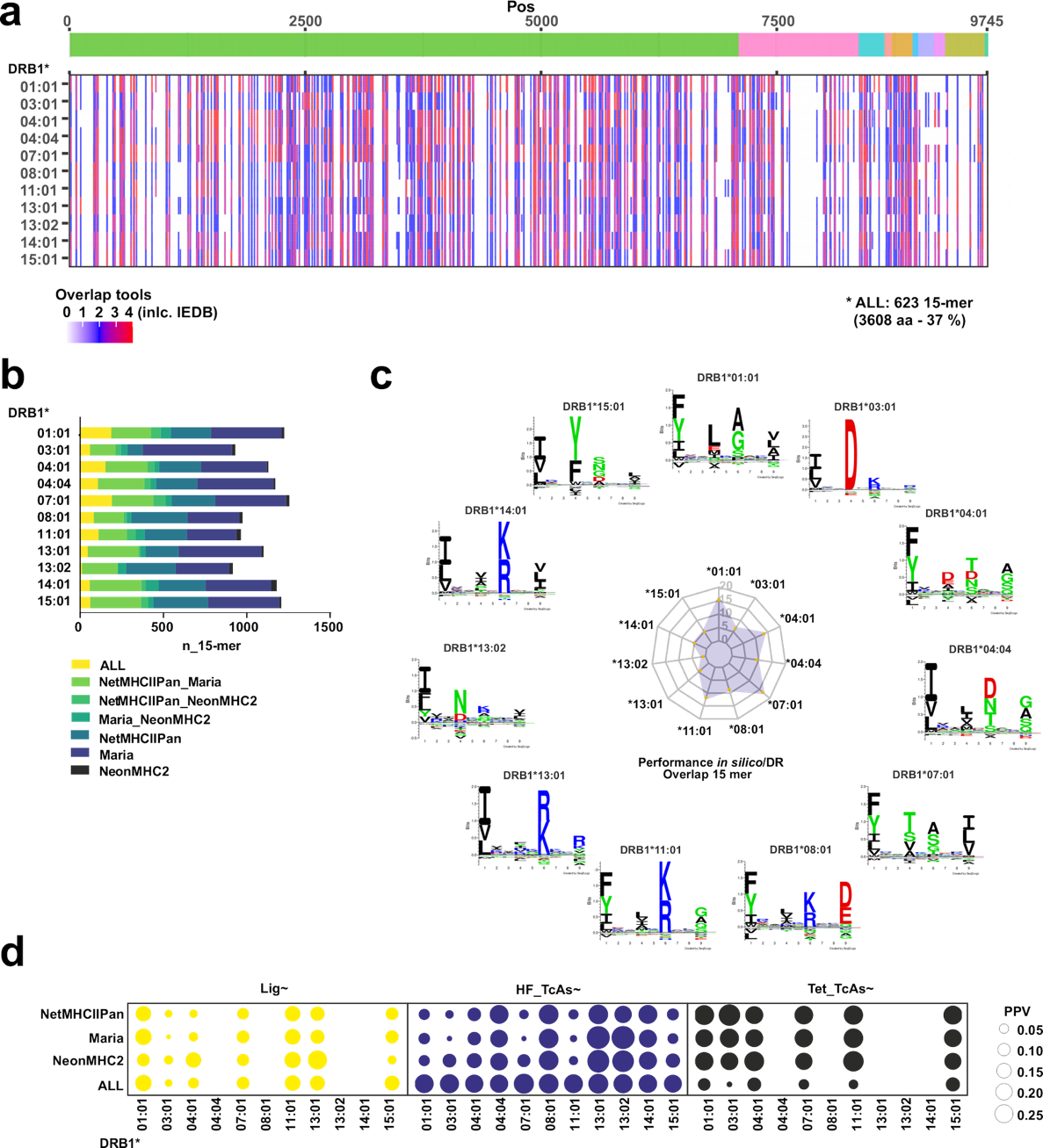
**

**Fig S2. Overview of the performance of the *in silico* tools used to predict binding and/or presentation by the selected set of DRB1- allotypes.** **a**. Summary of the overlap of the different tools plotted over the viral proteome (schematically presented on top) for each allotype selected. Hits are highlighted in colors on a white background (no hit) and the extent of overlap between tools is shown according to the scale shown in the legend. The number of 15-mers (n-15-mer) identified by each tool or combination of tools is shown as a cumulative bar chart. **b.** Global overlap (entire viral proteome) between the different predictors used for each allotype and shown as a bar-chart. **c.** Performance of the *in silico* prediction tools for each allotype, represented as the overlap of 15-mers over the total number of hits per allotype, on the middle radar-plot. On the outer part the binding motifs for each allotype are shown, as reported from eluted data available on NetMHCIIPan4.0. **d.** Summary of the positive predictive value for the models attained for predicting either Ligands (left), Prevalent T cell epitopes, (middle), and peptide-MHCII combinations described in tetrameric reagents (right) for each individual allotype and predictor.

**
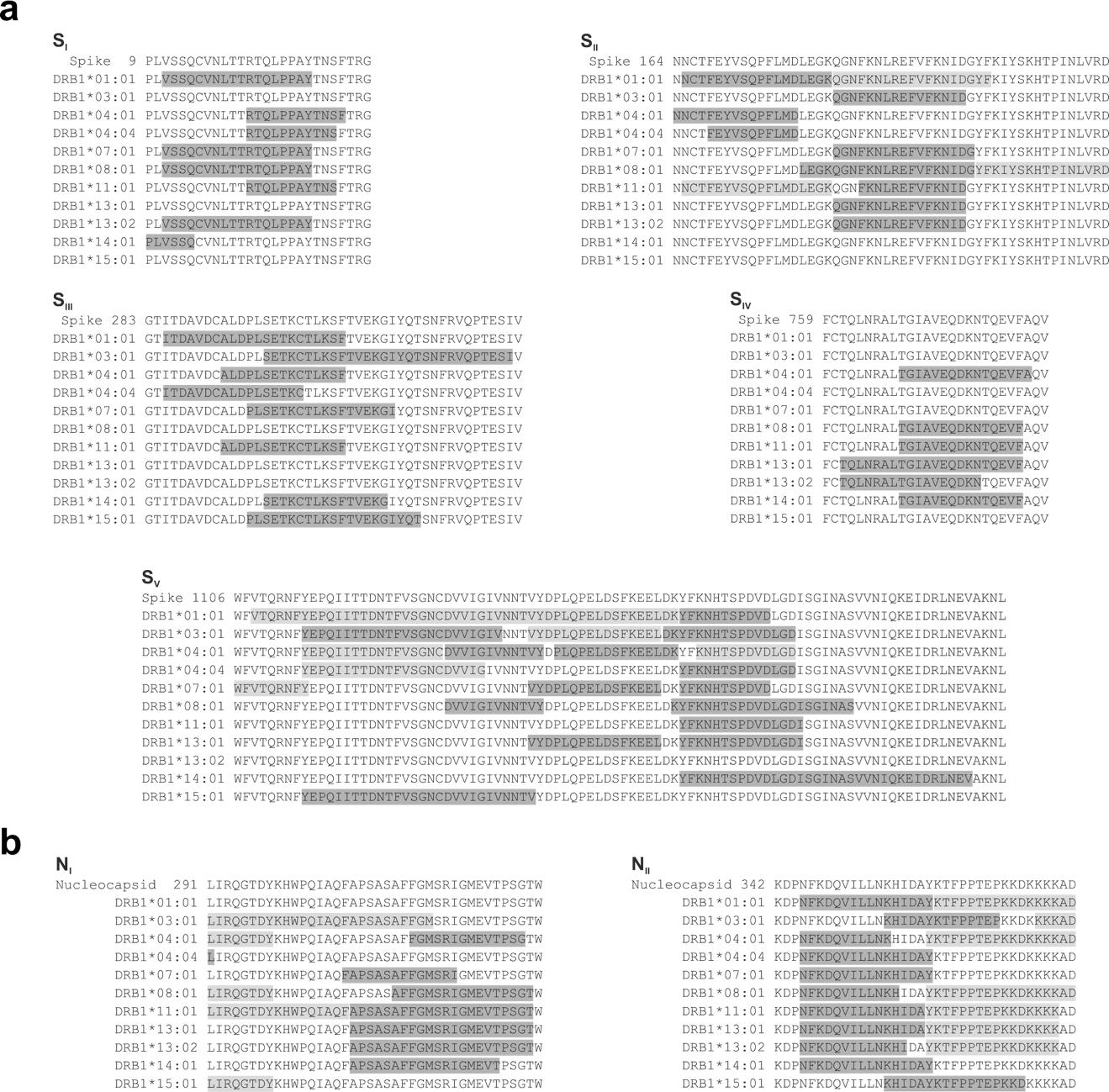
**

**Fig S3. Scheme of the antigenic regions recurrently selected by the DR allotypes tested by the reconstituted *in vitro* antigen processing system.** For each panel, the first line represents the antigenic sequence **a.** Spike, and **b.** Nucleocapsid, where the first amino acid´s position in the antigen sequence is provided. Subsequent lines indicate the peptides selected by each allotype (gray darkness correlate with the intensity of the peptides measured by MS).


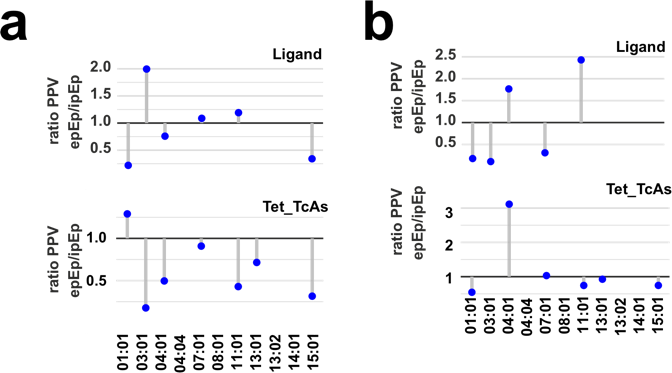


**Fig S4. Detailed analysis on the performance of the potential Epitope predictions. a.** Positive Predictive Value ratios for MS-derived (epEp) and *in silico* (ipEp) models for Ligand and Tetramer identifications for candidates for the **a.** Nucleocapsid, and **b.** Spike protein. A ratio lower than 1 refers to a better PPV of the *in silico* model whereas a ratio higher than 1 refers to a higher PPV of the MS-model for each allotype.


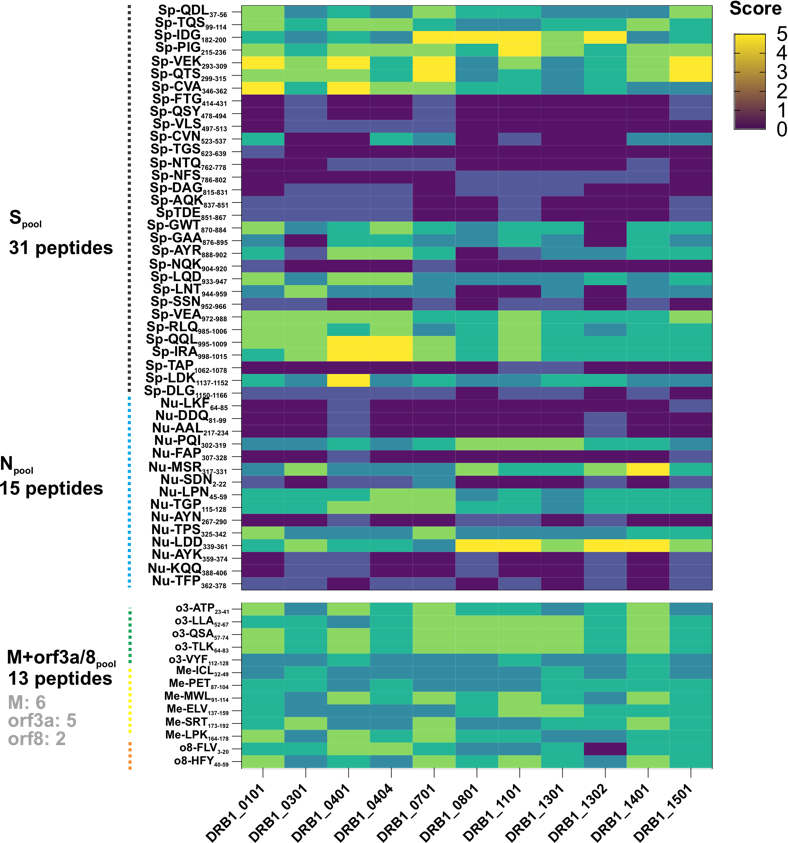


Fig S5. Overview of the minimalistic peptide pools (MPps). Scores indicate the number of times that they were found by any of the predictors used (see scale on the right side). Peptides are pooled by antigens and the total number of peptides of each pool is indicated. For the control peptide pool M+orf3a+orf8 the number of peptides derived from each antigen is indicated in gray. In the names of each individual peptide the name of the antigen and the first amino acid position is indicated.


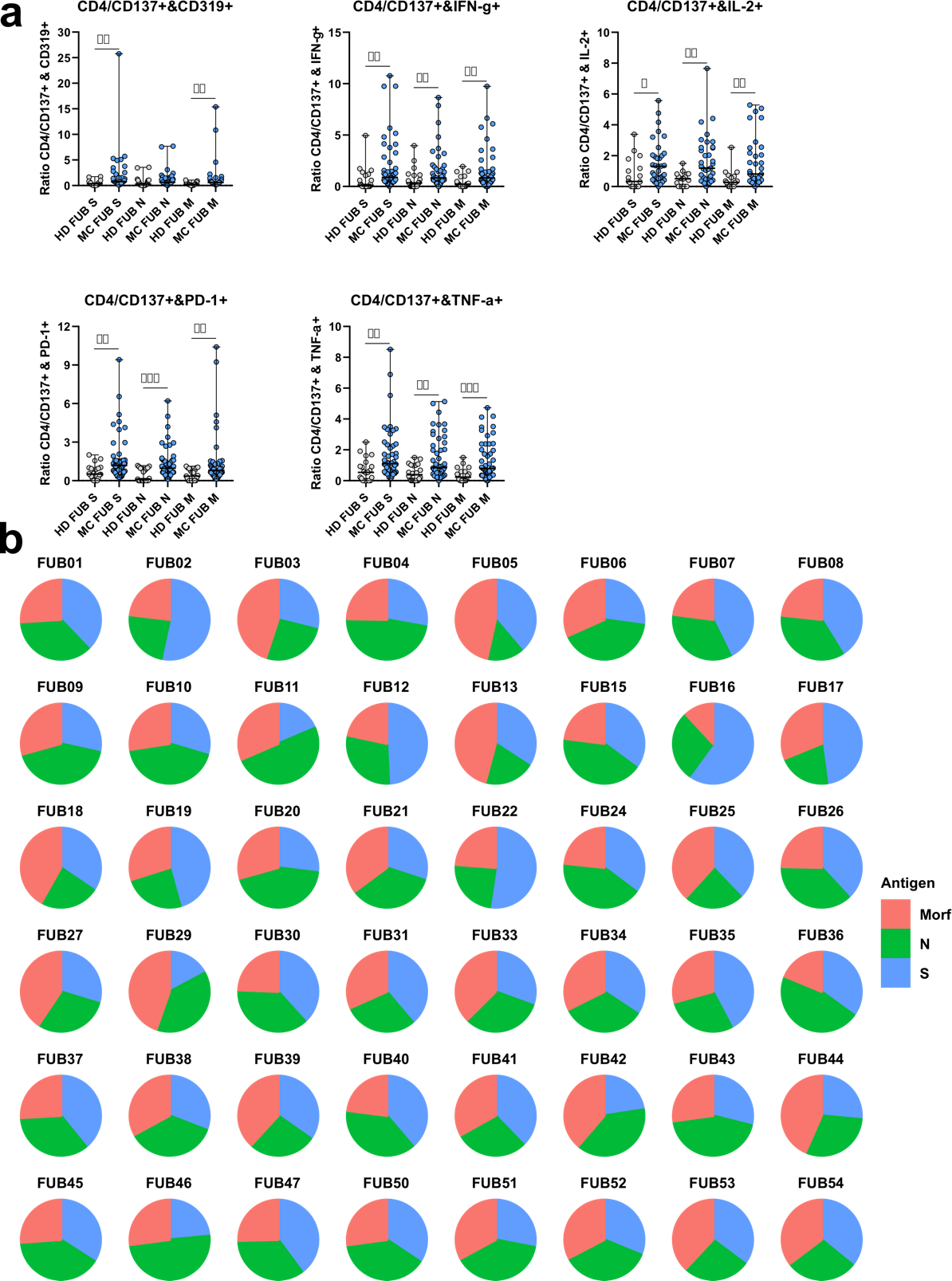


**Fig S6. Overview of the activation and profile of T cells responding to the broad coverage peptide panel. a.** Phenotype of Healthy donor (HD, n=24) and Post-COVID (MC n=48) responder cells to the pools used. Each group was tested for the activation via surface and intracellular cytokine stains upon stimulation with each peptide pool. The difference between the median of the responses was compared applying a non-parametric Mann-Whitney test, and the significance is reported as follows: *p < 0.05; **p < 0.01; ***p < 0.001; ****p < or = 0.0001. **b.** Contribution to the total response of each peptide pool in post-COVID individuals.


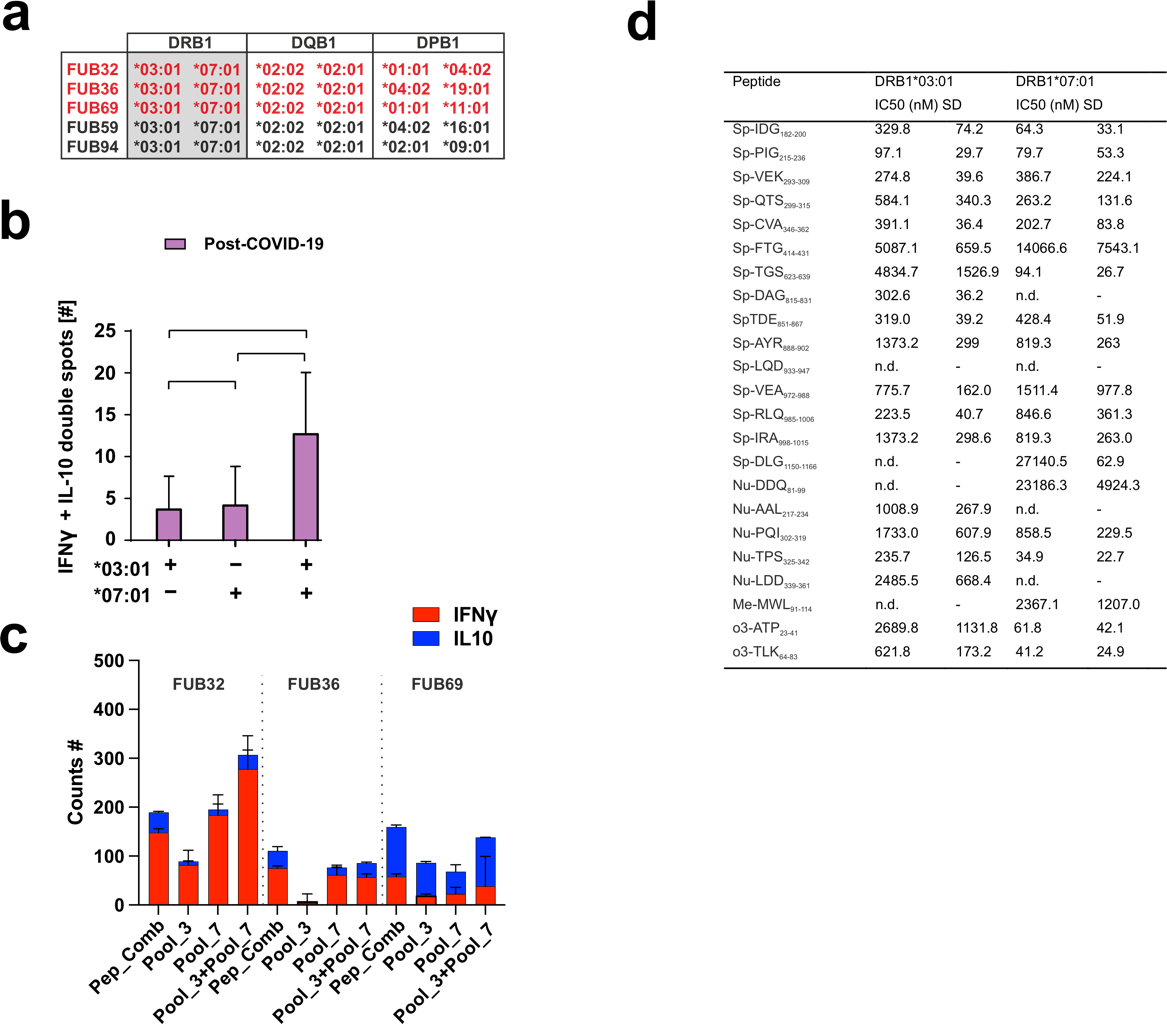


**Fig S7. Additional information on the experiments performed with HLA-matched individual’s cells. a.** Full HLA-types of the donors selected based on the HLA-DRB1* allotypes of interest (grey-shaded boxes). Individuals highlighted in red are those that recovered from SARS-CoV-2 infection. **b.** Number of clones secreting IFNγ and IL-10 for individuals recovered from SARS-CoV-2 infection when primed with peptide pools matching DRB1*03:01, DRB1*07:01 or both. Results show Average and SD of n=2 measurements on 3 individuals. Significance was defined using 1-way Annova with Holm-Sidak correction. * p<0.05, n.s. not significant. **c.** Overview of response achieved for each individual depending on the peptide pool used. Pep_Comb: sum of the responses achieved by the dual peptide combinations; Pool_3: peptide mixture from all those selected as preferential candidates for DRB1*03:01; Pool_7: peptide mixture from all those selected as preferential candidates for DRB1*07:01; Pool_3+Pool_7: the mixture of both peptide pools. The extent of the cytokine response measured for each cytokine is shown colored according to the legend. Measurements represent the average and SD for each individual measured in two technical replicates. **d.** Summary of the binding affinity measurements for the two DRB1*-allotypes. Peptide sequences are indicated as well as the Average IC50 values resulting from n=3 independent experiments measured in triplicates and SD are also indicated. n.d. refers to not determined.

**
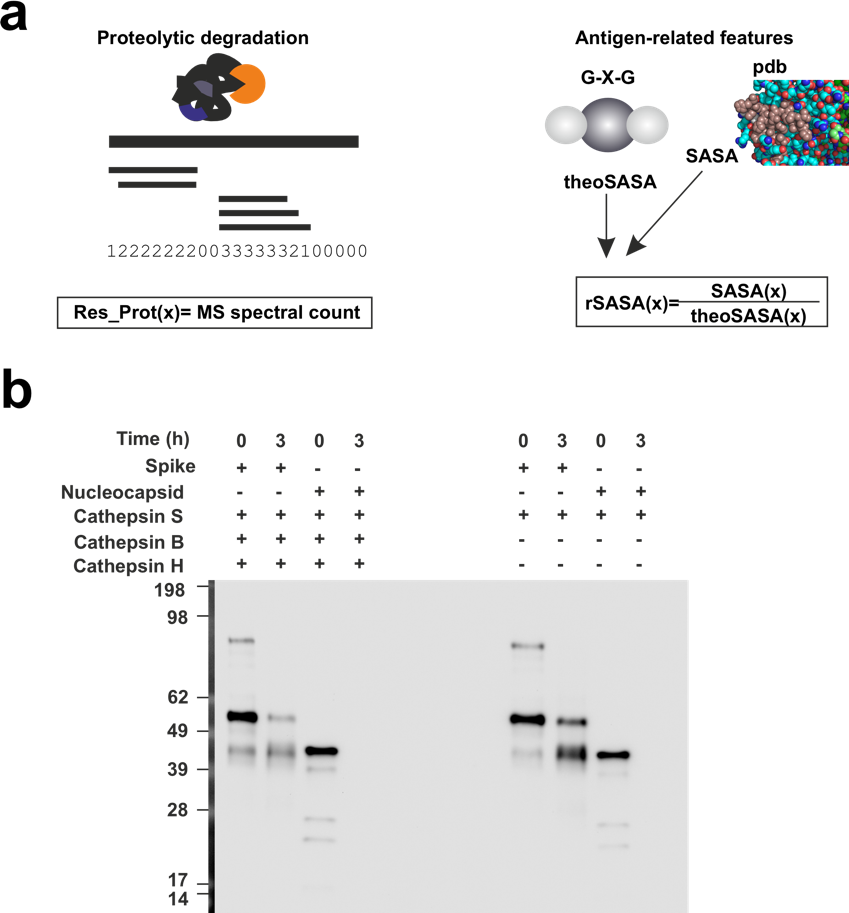
**

**Fig S8. Summary of the main antigen- and antigen-processing related features.** **a.** Scheme of the scoring system applied at the residue level for each of the features considered. Proteolytic degradation experiments were performed as indicated and spectral counts are considered to define Res_Prot and Sensitivity to proteases. Structure-related features were calculated from structural models available in the Zhang lab website (([https://zhanggroup.org//COVID-19/](https://zhanggroup.org/COVID-19/)) accessed on Sept. 2022 and coded with the corresponding measured values (rel_sasa). **b.** SDS-PAGE demonstrating the *in vitro* degradation of the antigens considered in the presence of the stated proteases for 1.5 and 3h (left), and the mapping of the measured peptides on a scheme of the corresponding antigens.


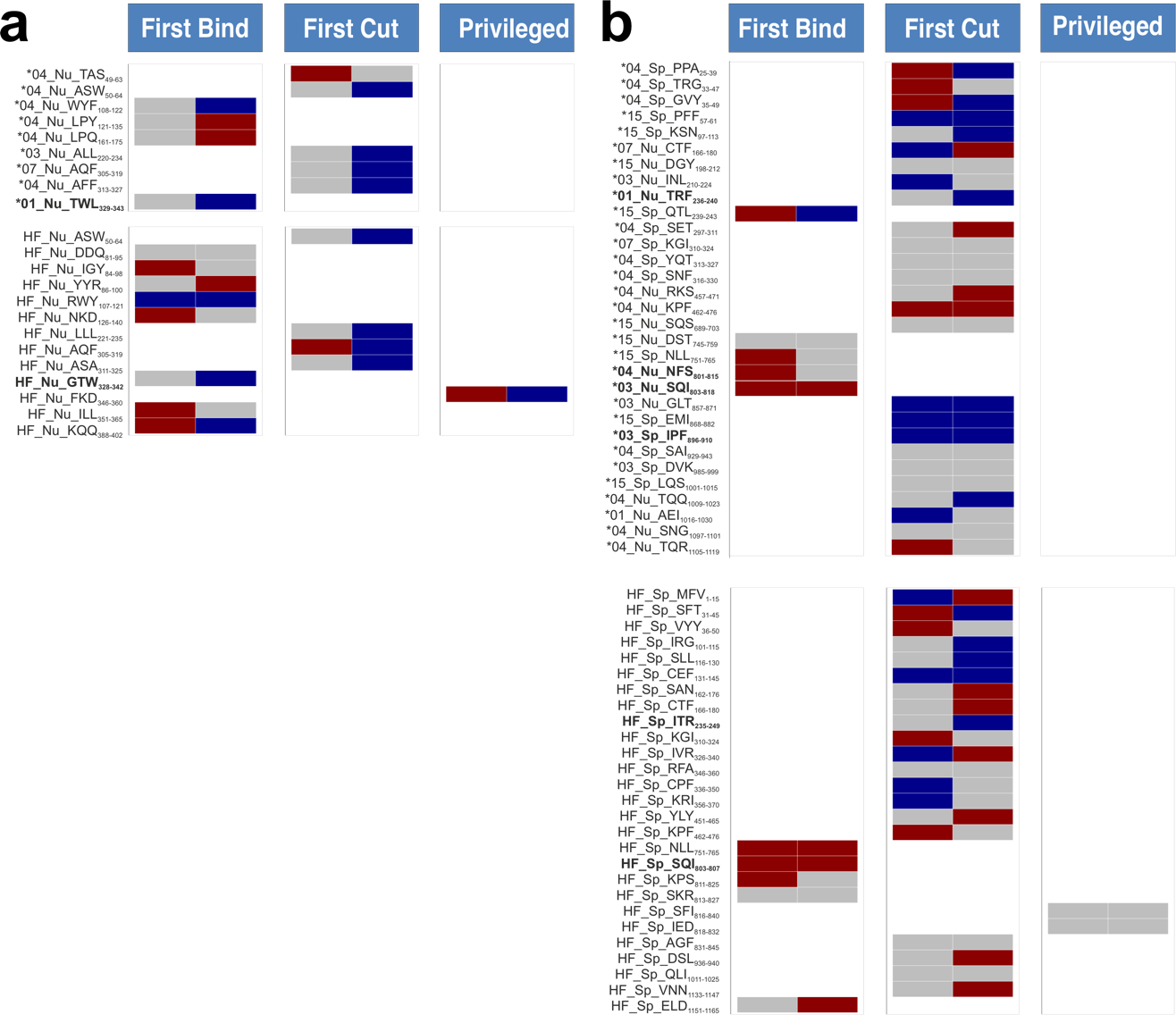


**Fig S9. Inferred peptide selection model for all known epitopes from the IEDB. a**. refers to the Nucleocapsid and **b**. for the Spike. In both cases antigen processing mechanism and antigen-intrinsic features of every known IEDB epitope according to our definition of Tet_TcAs (multimer identified) and HF_TcAs (positive epitopes tested in more than 10 individuals and triggering responses in at least 5) are shown. For Tet_TcAs entries, we indicate the main restriction associated to the peptide at a 2 digit resolution followed but a two characters code for each antigen (Nu for Nucleocapsid and Sp for Spike) and the first three amino acids of the entry followed by the positions covered. Peptides spanning same regions in Tet_TcAs and HF_TcAs and identified with more than one restriction as Tet_TcAs are shown in bold letters. Each model is inferred according to the parameter Sensitivity to proteases, then SASA and Res_Prot averaged values for each entry are considered. Random distributions of averaged feature values for peptides mapping to regions excluding HF_TcAs, Tet_TcAs and MPpN and MPpS are used as controls (note there is one distribution for each antigen consisting of 10 entries for the Nucleocapsid and 20 for the Spike protein). Wilcoxon rank test is used to compare medians of the control distribution with that of the corresponding epitope (significance levels: *p<0.05). Color coding reflects the direction and significance of the deviation from the control median: if the feature value for a given epitope was higher than the median of the corresponding random distribution, a red color was assigned; if it was lower, a blue color was used. Not statistically significant cases are represented in gray.


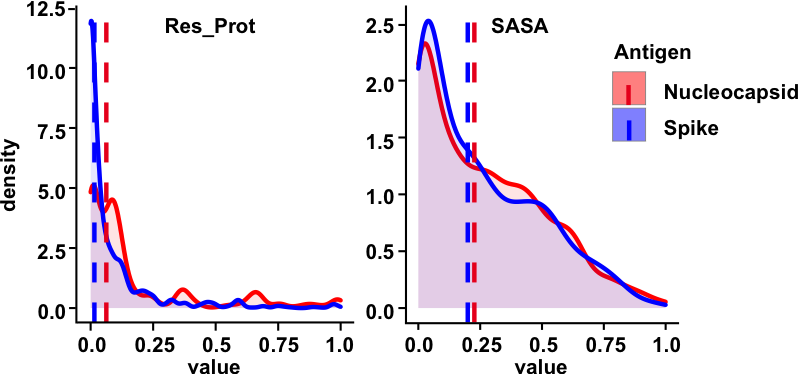


**Fig S10. Density plots of antigenic features related to antigen processing mechanisms**. Density plots for the values calculated for each of the parameters considered for each antigen. Wilcoxon-rank test indicates a significant different for all parameters considered n. Median values are shown as a dashed-colored line for each antigen and parameter according to the legend.

**Table S1. HLA-types of the donors recruited in this study**

|  | **sample ID** | **HLA-DRB1_1** | **HLA-DRB1_2** | **HLA-DQB1_1** | **HLA-DQB1_2** | **HLA-DPB1_1** | **HLA-DRP1_2** |
| --- | --- | --- | --- | --- | --- | --- | --- |
| **Healthy donors** | FUB068 | DRB1*01:02 | DRB1*14:54 | DQB1*05:03 | DQB1*05:01 | DPB1*04:01 | DPB1*04:01 |
|  | FUB080 | DRB1*03:01 | DRB1*15:01 | DQB1*02:01 | DQB1*06:02 | DPB1*04:01 | DPB1*04:01 |
|  | FUB097 | DRB1*01:01 | DRB1*07:01 | DQB1*03:03 | DQB1*05:01 | DPB1*04:01 | DPB1*04:01 |
|  | FUB107 | DRB1*11:04 | DRB1*15:01 | DQB1*03:01 | DQB1*06:02 | DPB1*04:01 | DPB1*04:01 |
|  | FUB105 | DRB1*12:01 | DRB1*15:01 | DQB1*03:01 | DQB1*06:02 | DPB1*04:02 | DPB1*04:01 |
|  | FUB075 | DRB1*03:01 | DRB1*07:01 | DQB1*02:01 | DQB1*03:03 | DPB1*04:01 | DPB1*04:01 |
|  | FUB078 | DRB1*01:01 | DRB1*13:03 | DQB1*03:01 | DQB1*05:01 | DPB1*03:01 | DPB1*11:01 |
|  | FUB103 | DRB1*08:01 | DRB1*09:01 | DQB1*02:02 | DQB1*04:02 | DPB1*03:01 | DPB1*17:01 |
|  | FUB092 | DRB1*01:01 | DRB1*07:01 | DQB1*03:03 | DQB1*05:01 | DPB1*03:01 | DPB1*04:01 |
|  | FUB065 | DRB1*04:04 | DRB1*04:01 | DQB1*03:02 | DQB1*03:02 | DPB1*02:01 | DPB1*04:01 |
|  | FUB079 | DRB1*07:01 | DRB1*14:06 | DQB1*03:03 | DQB1*03:01 | DPB1*04:02 | DPB1*10:01 |
|  | FUB083 | DRB1*11:03 | DRB1*13:03 | DQB1*03:01 | DQB1*03:01 | DPB1*04:02 | DPB1*124:01 |
|  | FUB093 | DRB1*03:01 | DRB1*11:01 | DQB1*02:01 | DQB1*03:01 | DPB1*04:01 | DPB1*04:01 |
|  | FUB061 | DRB1*01:01 | DRB1*01:01 | DQB1*05:01 | DQB1*05:01 | DPB1*04:02 | DPB1*04:02 |
|  | FUB062 | DRB1*01:01 | DRB1*13:01 | DQB1*05:01 | DQB1*06:03 | DPB1*02:01 | DPB1*03:01 |
|  | FUB100 | DRB1*07:01 | DRB1*12:01 | DQB1*02:02 | DQB1*03:01 | DPB1*02:01 | DPB1*04:02 |
|  | FUB099 | DRB1*04:01 | DRB1*07:01 | DQB1*02:02 | DQB1*03:01 | DPB1*03:01 | DPB1*04:01 |
|  | FUB071 | DRB1*04:03 | DRB1*15:01 | DQB1*03:02 | DQB1*06:02 | DPB1*03:01 | DPB1*04:01 |
|  | FUB069 | DRB1*01:01 | DRB1*15:01 | DQB1*05:01 | DQB1*06:02 | DPB1*02:01 | DPB1*04:02 |
|  | FUB108 | DRB1*11:01 | DRB1*15:01 | DQB1*03:01 | DQB1*06:02 | DPB1*04:02 | DPB1*04:01 |
|  | FUB084 | DRB1*08:06 | DRB1*13:01 | DQB1*06:02 | DQB1*06:03 | DPB1*02:01 | DPB1*17:01 |
|  | FUB091 | DRB1*03:01 | DRB1*13:02 | DQB1*02:01 | DQB1*06:04 | DPB1*04:01 | DPB1*04:01 |
|  | FUB081 | DRB1*13:01 | DRB1*15:01 | DQB1*06:02 | DQB1*06:03 | DPB1*02:01 | DPB1*04:01 |
|  | FUB087 | DRB1*01:01 | DRB1*13:02 | DQB1*05:01 | DQB1*06:04 | DPB1*03:01 | DPB1*13:01 |
|  | FUB094 | DRB1*03:01 | DRB1*07:01 | DQB1*02:02 | DQB1*02:01 | DPB1*04:02 | DPB1*16:01 |
|  | FUB082 | DRB1*03:01 | DRB1*04:01 | DQB1*02:01 | DQB1*03:02 | DPB1*01:01 | DPB1*03:01 |
|  | FUB086 | DRB1*04:04 | DRB1*13:02 | DQB1*03:02 | DQB1*06:04 | DPB1*03:01 | DPB1*06:01 |
|  | FUB073 | DRB1*09:01 | DRB1*11:01 | DQB1*03:03 | DQB1*03:01 | DPB1*04:02 | DPB1*20:01 |
|  | FUB106 | DRB1*13:01 | DRB1*13:02 | DQB1*06:04 | DQB1*06:03 | DPB1*02:01 | DPB1*03:01 |
|  | FUB090 | DRB1*16:01 | DRB1*16:01 | DQB1*05:02 | DQB1*05:02 | DPB1*04:02 | DPB1*10:01 |
|  | FUB085 | DRB1*07:01 | DRB1*14:54 | DQB1*02:02 | DQB1*05:03 | DPB1*04:01 | DPB1*16:01 |
|  | FUB066 | DRB1*01:01 | DRB1*11:01 | DQB1*03:01 | DQB1*05:01 | DPB1*04:01 | DPB1*09:01 |
|  | FUB089 | DRB1*03:01 | DRB1*10:01 | DQB1*02:01 | DQB1*05:01 | DPB1*01:01 | DPB1*04:01 |
|  | FUB098 | DRB1*08:01 | DRB1*15:01 | DQB1*04:02 | DQB1*06:02 | DPB1*02:01 | DPB1*04:01 |
|  | FUB088 | DRB1*01:01 | DRB1*15:01 | DQB1*05:01 | DQB1*06:02 | DPB1*04:01 | DPB1*04:01 |
|  | FUB104 | DRB1*01:02 | DRB1*13:01 | DQB1*05:01 | DQB1*06:03 | DPB1*04:02 | DPB1*17:01 |
|  | FUB076 | DRB1*11:04 | DRB1*13:01 | DQB1*03:01 | DQB1*03:01 | DPB1*04:02 | DPB1*10:01 |
|  | FUB102 | DRB1*01:01 | DRB1*13:01 | DQB1*05:01 | DQB1*06:03 | DPB1*02:01 | DPB1*06:01 |
|  | FUB063 | DRB1*07:01 | DRB1*13:01 | DQB1*02:02 | DQB1*06:03 | DPB1*04:02 | DPB1*14:01 |
|  | FUB095 | DRB1*04:01 | DRB1*13:01 | DQB1*03:01 | DQB1*06:03 | DPB1*04:01 | DPB1*04:01 |
|  | FUB077 | DRB1*08:04 | DRB1*15:01 | DQB1*03:01 | DQB1*06:02 | DPB1*02:01 | DPB1*09:01 |
|  | FUB101 | DRB1*01:01 | DRB1*11:01 | DQB1*03:01 | DQB1*05:01 | DPB1*03:01 | DPB1*04:01 |
|  | FUB074 | DRB1*04:01 | DRB1*09:01 | DQB1*03:03 | DQB1*03:01 | DPB1*04:02 | DPB1*04:01 |
|  | FUB070 | DRB1*03:01 | DRB1*11:01 | DQB1*02:01 | DQB1*03:01 | DPB1*04:01 | DPB1*04:01 |
|  | FUB072 | DRB1*04:01 | DRB1*07:01 | DQB1*02:02 | DQB1*03:02 | DPB1*03:01 | DPB1*17:01 |
|  | FUB067 | DRB1*08:01 | DRB1*16:01 | DQB1*04:02 | DQB1*05:02 | DPB1*03:01 | DPB1*23:01 |
|  | FUB096 | DRB1*03:01 | DRB1*13:01 | DQB1*02:01 | DQB1*06:03 | DPB1*02:01 | DPB1*04:02 |
|  | FUB064 | DRB1*03:01 | DRB1*13:03 | DQB1*02:01 | DQB1*03:01 | DPB1*01:01 | DPB1*02:01 |
|  | FUB55 | DRB1*04:08 | DRB1*07:01 | DQB1*02:02 | DQB1*03:01 | DPB1*01:01 | DPB1*02:01 |
|  | FUB56 | DRB1*04:01 | DRB1*07:01 | DQB1*02:02 | DQB1*03:01 | DPB1*04:01 | DPB1*04:01 |
|  | FUB57 | DRB1*03:01 | DRB1*09:01 | DQB1*02:02 | DQB1*02:01 | DPB1*02:01 | DPB1*17:01 |
|  | FUB58 | DRB1*11:04 | DRB1*15:01 | DQB1*03:01 | DQB1*06:02 | DPB1*04:01 | DPB1*04:01 |
|  | FUB59 | DRB1*03:01 | DRB1*07:01 | DQB1*02:02 | DQB1*02:01 | DPB1*02:01 | DPB1*09:01 |
|  | FUB60 | DRB1*07:01 | DRB1*14:54 | DQB1*02:02 | DQB1*05:03 | DPB1*03:01 | DPB1*14:01 |
|  | NAN | DRB1*01:01 | DRB1*03:01 | NAN | NAN | NAN | NAN |
| **Convalescent**  **SARS-CoV2^+^** | FUB01 | DRB1*03:01 | DRB1*04:01 | DQB1*02:01 | DQB1*03:02 | DPB1*02:01 | DPB1*04:01 |
|  | FUB02 | DRB1*04:01 | DRB1*11:03 | DQB1*03:01 | DQB1*03:01 | DPB1*04:01 | DPB1*06:01 |
|  | FUB03 | DRB1*11:01 | DRB1*11:01 | DQB1*03:01 | DQB1*03:01 | DPB1*02:01 | DPB1*04:01 |
|  | FUB04 | DRB1*03:01 | DRB1*11:01 | DQB1*02:01 | DQB1*03:01 | DPB1*01:01 | DPB1*03:01 |
|  | FUB05 | DRB1*04:04 | DRB1*16:01 | DQB1*03:02 | DQB1*05:02 | DPB1*02:01 | DPB1*04:01 |
|  | FUB06 | DRB1*01:01 | DRB1*04:01 | DQB1*03:02 | DQB1*05:01 | DPB1*04:01 | DPB1*04:01 |
|  | FUB07 | DRB1*04:01 | DRB1*15:01 | DQB1*03:02 | DQB1*06:02 | DPB1*04:01 | DPB1*04:01 |
|  | FUB08 | DRB1*03:01 | DRB1*07:01 | DQB1*02:02 | DQB1*02:01 | DPB1*01:01 | DPB1*11:01 |
|  | FUB09 | DRB1*03:01 | DRB1*11:01 | DQB1*02:01 | DQB1*03:01 | DPB1*02:01 | DPB1*04:01 |
|  | FUB10 | DRB1*03:01 | DRB1*03:01 | DQB1*02:01 | DQB1*02:01 | DPB1*04:01 | DPB1*13:01 |
|  | FUB11 | DRB1*03:01 | DRB1*08:01 | DQB1*02:01 | DQB1*04:02 | DPB1*02:01 | DPB1*04:01 |
|  | FUB12 | DRB1*01:02 | DRB1*01:01 | DQB1*05:01 | DQB1*05:01 | DPB1*04:01 | DPB1*13:01 |
|  | FUB13 | DRB1*07:01 | DRB1*07:01 | DQB1*03:03 | DQB1*03:03 | DPB1*02:01 | DPB1*04:01 |
|  | FUB14 | DRB1*03:01 | DRB1*15:02 | DQB1*02:01 | DQB1*06:01 | DPB1*02:01 | DPB1*09:01 |
|  | FUB15 | DRB1*03:01 | DRB1*11:04 | DQB1*02:01 | DQB1*03:01 | DPB1*04:01 | DPB1*04:02 |
|  | FUB16 | DRB1*04:01 | DRB1*15:01 | DQB1*03:02 | DQB1*06:02 | DPB1*01:01 | DPB1*04:01 |
|  | FUB17 | DRB1*11:04 | DRB1*16:01 | DQB1*03:01 | DQB1*05:02 | DPB1*03:01 | DPB1*04:01 |
|  | FUB18 | DRB1*15:01 | DRB1*16:01 | DQB1*05:02 | DQB1*06:02 | DPB1*04:01 | DPB1*04:01 |
|  | FUB19 | DRB1*15:01 | DRB1*16:01 | DQB1*05:02 | DQB1*06:02 | DPB1*03:01 | DPB1*04:01 |
|  | FUB20 | DRB1*10:01 | DRB1*13:01 | DQB1*05:01 | DQB1*06:03 | DPB1*04:01 | DPB1*04:01 |
|  | FUB21 | DRB1*04:04 | DRB1*07:01 | DQB1*02:02 | DQB1*03:02 | DPB1*04:01 | DPB1*06:01 |
|  | FUB22 | DRB1*04:01 | DRB1*15:01 | DQB1*03:01 | DQB1*06:02 | DPB1*04:01 | DPB1*04:01 |
|  | FUB23 | DRB1*07:01 | DRB1*15:01 | DQB1*02:02 | DQB1*06:02 | DPB1*04:01 | DPB1*11:01 |
|  | FUB24 | DRB1*01:01 | DRB1*15:01 | DQB1*05:01 | DQB1*06:02 | DPB1*04:01 | DPB1*04:01 |
|  | FUB25 | DRB1*11:01 | DRB1*11:04 | DQB1*03:01 | DQB1*03:01 | DPB1*04:01 | DPB1*04:02 |
|  | FUB26 | DRB1*03:01 | DRB1*08:01 | DQB1*02:01 | DQB1*03:01 | DPB1*01:01 | DPB1*04:01 |
|  | FUB27 | DRB1*03:01 | DRB1*11:01 | DQB1*02:01 | DQB1*03:01 | DPB1*04:02 | DPB1*06:01 |
|  | FUB28 | DRB1*03:01 | DRB1*11:04 | DQB1*02:01 | DQB1*03:01 | DPB1*02:01 | DPB1*02:01 |
|  | FUB29 | DRB1*04:02 | DRB1*11:01 | DQB1*03:02 | DQB1*03:01 | DPB1*03:01 | DPB1*06:01 |
|  | FUB30 | DRB1*09:01 | DRB1*11:01 | DQB1*03:03 | DQB1*03:01 | DPB1*04:01 | DPB1*04:01 |
|  | FUB31 | DRB1*04:01 | DRB1*13:01 | DQB1*03:02 | DQB1*06:03 | DPB1*04:01 | DPB1*19:01 |
|  | FUB32 | DRB1*03:01 | DRB1*07:01 | DQB1*02:02 | DQB1*02:01 | DPB1*01:01 | DPB1*04:02 |
|  | FUB33 | DRB1*11:01 | DRB1*16:01 | DQB1*03:01 | DQB1*05:02 | DPB1*04:01 | DPB1*04:02 |
|  | FUB34 | DRB1*01:01 | DRB1*03:01 | DQB1*02:01 | DQB1*05:01 | DPB1*04:01 | DPB1*04:02 |
|  | FUB35 | DRB1*11:01 | DRB1*15:01 | DQB1*03:01 | DQB1*06:03 | DPB1*02:01 | DPB1*03:01 |
|  | FUB36 | DRB1*03:01 | DRB1*07:01 | DQB1*02:02 | DQB1*02:01 | DPB1*04:02 | DPB1*19:01 |
|  | FUB37 | DRB1*13:02 | DRB1*15:01 | DQB1*06:04 | DQB1*06:02 | DPB1*02:01 | DPB1*04:01 |
|  | FUB38 | DRB1*03:01 | DRB1*15:01 | DQB1*02:01 | DQB1*06:02 | DPB1*01:01 | DPB1*09:01 |
|  | FUB39 | DRB1*07:01 | DRB1*13:01 | DQB1*03:03 | DQB1*06:03 | DPB1*04:01 | DPB1*04:01 |
|  | FUB40 | DRB1*04:04 | DRB1*04:01 | DQB1*03:02 | DQB1*03:02 | DPB1*04:01 | DPB1*04:01 |
|  | FUB41 | DRB1*03:01 | DRB1*04:01 | DQB1*02:01 | DQB1*03:02 | DPB1*04:02 | DPB1*05:01 |
|  | FUB42 | DRB1*13:01 | DRB1*15:01 | DQB1*06:02 | DQB1*06:03 | DPB1*04:01 | DPB1*04:02 |
|  | FUB43 | DRB1*08:01 | DRB1*15:01 | DQB1*04:02 | DQB1*06:02 | DPB1*02:01 | DPB1*04:01 |
|  | FUB44 | DRB1*04:01 | DRB1*13:02 | DQB1*03:02 | DQB1*06:04 | DPB1*02:01 | DPB1*04:01 |
|  | FUB45 | DRB1*11:01 | DRB1*13:02 | DQB1*03:01 | DQB1*06:09 | DPB1*02:01 | DPB1*04:02 |
|  | FUB46 | DRB1*03:01 | DRB1*16:01 | DQB1*02:01 | DQB1*05:02 | DPB1*01:01 | DPB1*04:02 |
|  | FUB47 | DRB1*04:05 | DRB1*04:01 | DQB1*02:02 | DQB1*03:01 | DPB1*03:01 | DPB1*04:01 |
|  | FUB48 | DRB1*01:02 | DRB1*08:01 | DQB1*04:02 | DQB1*05:01 | DPB1*04:01 | DPB1*04:01 |
|  | FUB49 | DRB1*11:01 | DRB1*11:01 | DQB1*03:01 | DQB1*03:01 | DPB1*02:01 | DPB1*03:01 |
|  | FUB50 | DRB1*07:01 | DRB1*11:01 | DQB1*03:03 | DQB1*03:01 | DPB1*16:01 | DPB1*52:01 |
|  | FUB51 | DRB1*11:02 | DRB1*13:02 | DQB1*03:19 | DQB1*06:04 | DPB1*03:01 | DPB1*15:01 |
|  | FUB52 | DRB1*11:01 | DRB1*16:01 | DQB1*03:01 | DQB1*05:02 | DPB1*02:01 | DPB1*04:02 |
|  | FUB53 | DRB1*01:01 | DRB1*15:01 | DQB1*05:01 | DQB1*05:02 | DPB1*03:01 | DPB1*03:01 |
|  | FUB54 | DRB1*07:01 | DRB1*14:01 | DQB1*02:02 | DQB1*06:03 | DPB1*03:01 | DPB1*04:01 |

**Table S1**. Naming and HLA-type of the 109 individuals recruited for this study.

**Table S2. T cell assay data summary as retrieved from the IEDB**

| Authors  et al. | Count |  | HF  TcAs | Lig | Antigens Tested | Antigen Hits | Ref |
| --- | --- | --- | --- | --- | --- | --- | --- |
| Alison Tarke | 778 |  |  | Yes | ALL | Orf1ab, Orf3a, Orf6, Orf7a, Orf7b, Orf8, Orf10, S, N, M | [1] |
| Hendrik Karsten | 584 |  | Yes |  | S | S | [2] |
| Janna Heide | 431 |  | Yes |  | E, M, N | E, M, N | [3] |
| Jose Mateus | 258 |  |  | Yes | ALL | Orf1ab, Orf3a, Orf6, Orf7a, Orf8, S, N, M, E | [4] |
| Alexandra M Johansson | 252 |  |  |  | S, N, M | S, N, M | [5] |
| Julia Lang-Meli | 128 |  |  |  | S | S | [6] |
| Johan Verhagen | 119 |  |  |  | S, N | S, N | [7] |
| Kerry J. Laing Ph.D. | 78 |  |  |  | n.a. | S | *n.a.* |
| Annika Nelde | 68 |  |  | Yes | ALL | Orf1ab, S, N, M, E, Orf6, Of7a, Orf8, Orf10 | [8] |
| Aleksei Titov | 61 |  | Yes |  | Orfa1b, S, N, M, Orf3 | S, N, M | [9] |
| Jun Siong Low | 51 |  |  |  | S, N | S | [10] |
| Swayam Prakash | 47 |  | Yes | Yes | ALL (int desc pred) | Orf1ab, Orf6, Orf7a, Orf7b, Orf8, S, N, M, E | [11] |
| Xiaoxiao Jin | 44 |  |  |  | n.c | Orf1ab, S, N, M, E | [12] |
| Bezawit A Woldemeskel | 41 |  |  |  | S | S | [13] |
| Peter J Eggenhuizen | 40 |  | Yes |  | Orf1ab | Orf1ab (cross reactive BGC vaccine) | [14] |
| Ryan W Nelson | 36 |  | Yes |  | S, N | S, N | [15] |
| Maarten E Emmelot | 34 |  |  |  | S | S | [16] |
| Hans-Georg Rammensee | 25 |  |  |  | S, M, N, E | S, M, N, E | [17] |
| Laurent Bartolo | 25 |  | Yes |  | S, N, Orf8 | S, N, Orf8 | [18] |
| Michael D Keller | 24 |  |  |  | S, M, N, E, | S, M, N | [19] |
| Jonah Lin | 24 |  |  |  | S, M, N, E | S, N, M | [20] |
| Jonas S Heitmann | 22 |  | Yes |  | Orf8, S, M, N, E | S, M, N, E | [21] |
| Swapnil Mahajan | 20 |  |  |  | S | S | [22] |
| Mateus V de Castro | 20 |  |  |  | Orf1ab, S, Orf3a, N, Orf8, Orf6, E, Orf7a, M | Orf1ab, S, Orf3a, N, Orf8, Orf6, E, Orf7a, M | [23] |
| Tatjana Bilich | 19 |  |  |  | ALL | Orf1ab, S, Orf3a, N, Orf8, Orf6, E, Orf7a, M, Orf0 | [24] |
| Lucie Loyal | 19 |  | Yes | Yes | ALL (pool) | S | [25] |
| Nina Le Bert | 18 |  |  |  | Orf1ab, N* | Orf1ab, N* (M and S) | [26] |
| Xiuyuan Lu | 17 |  |  |  | S | S | [27] |
| Katarzyna Piadel | 16 |  |  |  | Orf1ab, N, E, Orf7a, M | Orf1ab, N, E, Orf7a, M | [28] |
| Philip A Mudd | 16 |  |  |  | S | S | [29] |
| Yuri Poluektov | 15 |  |  |  | Orf1ab, S, E, N | Orf1ab, S, E, N | [30] |
| Archana Panikkar | 15 |  |  |  | Orf3a, N, M, S | S, N, M | [31] |
| Luo Li | 12 |  |  |  | S | S | [32] |
| Yunwen Zhang | 12 |  |  |  | S | S | [33] |
| Yanchun Peng | 11 |  |  |  | All except Orf1ab | S, N, Orf7a, M | [34] |
| Kathleen M Wragg | 9 |  |  |  | S | S | [35] |
| Mikhail V Pogorelyy | 9 |  |  |  | S, N, M | S, N, M | [36] |
| Yipeng Ma | 8 |  |  | Yes | ALL (not tested) | Orf6, Orf10, M | [37] |
| Franz-Josef Obermair | 8 |  |  |  | ALL | S, N, M | [38] |
| Juan Zhao | 7 |  |  |  | S, N | S, N | [39] |
| Arbor G Dykema | 4 |  |  |  | S, N | S | [40] |
| Leo Swadling | 4 |  |  |  | Orf1ab | Orf1ab | [41] |
| Valerie Oberhardt | 3 |  |  |  | S | S | [42] |
| Thushan I de Silva | 2 |  |  |  | S | S | [43] |
| Katja G Schmidt | 1 |  |  |  | N | N | [44] |
| Wei Hu | 1 |  |  |  | S, E, M, N | M | [45] |
| Louise C Rowntree | 1 |  |  |  | S | S | [46] |

**Table S2**. Summary of the T cell assay data retrieved from the IEDB on September 2022. First author of the publication (first column) and entry of the bibliographic reference (last column). “Count” refers to the number of entries yielding positive responses and “HF_TcAs” (Yes) indicate that the study identifies entries that yield positive responses in at least half the tested (more than 10) individuals. “Lig” (Yes) states whether the study compiles ligand information and “Antigen Tested” and “Antigen Hits” refer to the orfs that where considered and yielded responses in the corresponding study.

**Table S3. Ligand Table summary as retrieved from the IEDB**

**Table S3.** Summary of the Ligand data retrieved from the IEDB on September 2022. First author of the publication (first column) and entry of the bibliographic reference (last column). “Type of Assay” indicate whether the entry was defined upon elution from MHC molecules in an immunopeptidome analysis or using synthetic peptides, and the type of assay that was conducted is referenced in “Experimental setup”. “Antigen Tested” refers to the source(s) of peptides that were considered in the study and “n_peptides/nested” indicates whether the study considers series of nested peptides. “Mono“ and “Multi“ refer to the background in which ligand binding is assessed, single allotypes in isolation or derived from cellular models in which several allotypes are present. “MHCII restrictions” tested at the molecular level and resulting number of “IEDB” entries. In case of “positive assays”, the number of entries with “semi-“ or “quantitative” information is referred.

| Authors | Type of assay | Experimental setup | Antigen tested | Mono | Multi | MHCII restriction | IEDB | n_pept/  nested | Positive assay (semiquant) | Positive assay (quantitative) | Ref |
| --- | --- | --- | --- | --- | --- | --- | --- | --- | --- | --- | --- |
| Nagler | Peptidome | Overexpression viral proteins | E, M, N, nsp6 | 1 | 2 | DR | 10 | 5 | - | - | [47] |
| Knierman | Peptidome | Mo-DCs + Protein | Spike | 0 | 9 | DR/DP | 604 | 73 | - | - | [48] |
| Parker | Peptidome | Mo-DCs + Protein | Spike | 0 | 5 | DR/DP | 161 | 29 | - | - | [49] |
| Heide | Peptides | Recombinant MHCII | E, M, N | 21 | 0 | DP/DQ/DR | 259 | 14 | - | 97 (500nM) | *(50)* |
| Tarke | Peptides | Recombinant MHCII | ALL | 15 | 0 | DR | 486 | 49 | - | 221 (500nM) | [1] |
| Liu | Peptides | Yeast-display MHCII | Spike | 1 | 0 | DR | 91 | 74 | 45 | - | [50] |
| Poluektov | Peptides/  Predicted | Recombinant MHCII | ? | 3 | 0 | DR | 53 | 19 | - | 53 | [30] |
| Prachar | Peptides/  Predicted | NeoScreen/  Recombinant MHCII | ? | 1 | 0 | DR | 22 | 94 | - | - | [51] |

**Table S4. Overview of the reconstituted *in vitro* antigen processing system**

|  | MS (Identifications) | | | | | | |
| --- | --- | --- | --- | --- | --- | --- | --- |
| DRB1  allele | -Cont  -Rev | -8aa | Unique  pep | Unique  prot | Cons  pep | Prot | Avg Size |
| *01:01 | 4639 | 4627 | 625 | 24 | 106 | 18 | 18.188 |
| *03:01 | 6210 | 6194 | 757 | 32 | 100 | 25 | 17.43 |
| *04:01 | 10049 | 9981 | 977 | 30 | 94 | 21 | 17.361 |
| *04:04 | 1546 | 1540 | 287 | 16 | 53 | 10 | 17.056 |
| *07:01 | 1480 | 1474 | 236 | 18 | 57 | 16 | 17.228 |
| *08:01 | 2731 | 2725 | 310 | 20 | 63 | 16 | 17.714 |
| *11:01 | 4437 | 4420 | 575 | 30 | 89 | 21 | 17.932 |
| *13:01 | 9070 | 9062 | 518 | 36 | 58 | 16 | 17.810 |
| *13:02 | 3480 | 3468 | 393 | 32 | 66 | 21 | 17.318 |
| *14:01 | 1589 | 1588 | 178 | 12 | 37 | 10 | 18.297 |
| *15:01 | 1785 | 1783 | 374 | 26 | 73 | 24 | 17.205 |

**Table S4.** Summary of the *in vitro* reconstituted antigen processing system. Raw files were used to identify peptides present in the samples on MaxQuant using a customized database that included the entire SARS-Cov-2 protein and all recombinant molecules used. All raw files were processed on the same search and the total No. IDs. was 102081, which after filtering contaminants and reverse identification (“-Cont, -Rev”) was reduced to 96689. Peptides of sizes smaller than 8 amino acids were also removed for the analysis (“-8 aa”) and when considering a PEP cutoff of 0.01 was reduced to 47016. “Unique pep” (peptides) or “prot” (proteins) identified are indicated, as well as the number of “Cons pep” (series of nested peptides) and the number of “Prot” (proteins) they derive from. Note as well that the “Avg (average) size” of the eluted peptides is uniformly distributed.

**Table S5. Peptides tested**

|  | Name | Sequence | Source | Start | End | Size | Strain mut |
| --- | --- | --- | --- | --- | --- | --- | --- |
| 1 | Sp-QDL_37-56_ | QDLFLPFFSNVTWFH**A**IHVS | Spike | 37 | 56 | 19 | **η** |
| 2 | Sp-TQS_99-114_ | TQSLLIVNNATNVVIK | Spike | 99 | 114 | 15 |  |
| 3 | Sp-IDG_182-200_ | IDGYFKIY**S**KHTPINLVRD | Spike | 182 | 200 | 18 |  |
| 4 | Sp-PIG_215-236_ | **P**IGINITRFQTLLALHRSYLTP | Spike | 215 | 236 | 21 | **Β,** |
| 5 | Sp-VEK_293-309_ | VEKGIYQTSNFRVQPTE | Spike | 293 | 309 | 16 |  |
| 6 | Sp-QTS_299-315_ | QTSNFRVQPTESIVRFP* | Spike | 299 | 315 | 16 |  |
| 7 | Sp-CVA_346-362_ | **C**VADYSVLYNSASFSTF | Spike | 346 | 362 | 16 | **μ** |
| 8 | Sp-FTG_414-431_ | FTGCVIAWNSNNLDSKVG | Spike | 414 | 431 | 17 |  |
| 9 | Sp-QSY_478-494_ | **Q**SYGFQ**P**TNGVGYQPYR | Spike | 478 | 494 | 16 | **δ,o/β,γ,η,к,o,μ** |
| 10 | Sp-VLS_497-513_ | V**L**SF**E**LLH**A**PATVCGPK | Spike | 497 | 513 | 16 | o/α,**β,γ**,o,**θ**,**μ/o** |
| 11 | Sp-CVN_523-537_ | CVNFNFNGLTGTGVL | Spike | 523 | 537 | 14 |  |
| 12 | Sp-TGS_623-639_ | TGSNVFQTRAGCLIGAE | Spike | 623 | 639 | 16 |  |
| 13 | Sp-NTQ_762-778_ | NT**Q**EVFAQVKQIYKTPP | Spike | 762 | 778 | 16 | o |
| 14 | Sp-NFS_786-802_ | NFSQILPDPS**K**PSKRSF | Spike | 786 | 802 | 16 |  |
| 15 | Sp-DAG_815-831_ | DAGFIKQYGDCLGDIAA | Spike | 815 | 831 | 16 |  |
| 16 | Sp-AQK_837-851_ | AQKFNGLTVLPPLLT | Spike | 837 | 851 | 14 |  |
| 17 | SpTDE_851-867_ | TDEMIAQY**T**SALLAGTI | Spike | 851 | 867 | 16 | ***o, λ** |
| 18 | Sp-GWT_870-884_ | GWTFGAGAALQI**P**FA | Spike | 870 | 884 | 14 |  |
| 19 | Sp-GAA_876-895_ | GAALQI**P**FAMQMAYRFNGIG | Spike | 876 | 895 | 19 | **η*** |
| 20 | Sp-AYR_888-902_ | AYRFNGIGVTQNVLY | Spike | 888 | 902 | 14 | **η** |
| 21 | Sp-NQK_904-920_ | NQKLIANQFNSAIGKIQ | Spike | 904 | 920 | 16 |  |
| 22 | Sp-LQD_933-947_ | LQDVVNQNAQALNTL | Spike | 933 | 947 | 14 |  |
| 23 | Sp-LNT_944-959_ | LNTLVK**Q**LSS**N**FGAIS | Spike | 944 | 959 | 15 | **δ,o,μ** |
| 24 | Sp-SSN_952-966_ | SS**N**FGAISSVLNDIL | Spike | 952 | 966 | 14 | o |
| 25 | Sp-VEA_972-988_ | VEAEVQIDR**LI**TGRLQS | Spike | 972 | 988 | 16 | o, α, |
| 26 | Sp-RLQ_985-1006_ | RLQSLQTYVTQQLIRAAEIRAS | Spike | 985 | 1006 | 21 |  |
| 27 | Sp-QQL_995-1009_ | QQLIRAAEIRASANL | Spike | 995 | 1009 | 14 |  |
| 28 | Sp-IRA_998-1015_ | IRAAEIRASANLAATKMS | Spike | 998 | 1015 | 17 |  |
| 29 | Sp-TAP_1062-1078_ | TAPAICHDG**K**AHFPREG | Spike | 1062 | 1078 | 16 |  |
| 30 | Sp-LDK_1137-1152_ | LDKYFKNHTSPDVDLG | Spike | 1137 | 1152 | 15 |  |
| 31 | Sp-DLG_1150-1166_ | DLGDISGINASVVNIQK | Spike | 1150 | 1166 | 16 |  |
| 38 | Nu-SDN_2-22_ | **SD**NGPQNQRN**AP**RITFGGPSD | Nucleocapsid | 2 | 22 | 20 | α,*, **η, λ** |
| 39 | Nu-LPN_45-59_ | LPNNTASWFTALTQH | Nucleocapsid | 45 | 59 | 14 |  |
| 32 | Nu-LKF_64-85_ | LKFPRGQGVPINTNSS**P**DDQIG | Nucleocapsid | 64 | 85 | 21 |  |
| 33 | Nu-DDQ_81-99_ | DDQIGYYRRATRRIRGGDG | Nucleocapsid | 81 | 99 | 18 |  |
| 40 | Nu-TGP_115-128_ | TGPE**A**GLPYGANKD | Nucleocapsid | 115 | 128 | 13 |  |
| 34 | Nu-AAL_217-234_ | AALALLLLDRLNQLESK**M** | Nucleocapsid | 217 | 234 | 17 | α, **ζ** |
| 41 | Nu-AYN_267-290_ | AYNVTQAFGRRGPEQTQGNFGDQE | Nucleocapsid | 267 | 290 | 23 |  |
| 35 | Nu-PQI_302-319_ | PQIAQFAPSASAFFGMSR | Nucleocapsid | 302 | 319 | 17 |  |
| 36 | Nu-FAP_307-328_ | FAPSASAFFGMSRIGMEVTPSG | Nucleocapsid | 307 | 328 | 21 |  |
| 37 | Nu-MSR_317-331_ | MSRIGMEVTPSGTWL | Nucleocapsid | 317 | 331 | 14 |  |
| 42 | Nu-TPS_325-342_ | TPSGTWLTYTGAIKLDDK | Nucleocapsid | 325 | 342 | 17 |  |
| 43 | Nu-LDD_339-361_ | LDDKDPNFKDQVILLNKHIDAYK | Nucleocapsid | 339 | 361 | 22 |  |
| 44 | Nu-AYK_359-374_ | AYKTFPPTEPKKDKKK | Nucleocapsid | 359 | 374 | 15 |  |
| 45 | Nu-KQQ_388-406_ | KQQTVTLLPAADLDDFSKQ | Nucleocapsid | 388 | 406 | 18 |  |
| 46 | Nu-TFP_362-378_ | TFPPTEPKKDKKKKA*D*E* | Nucleocapsid | 362 | 378 | 16 |  |
| 47 | Me-ICL_32-49_ | ICLLQFAYANRNRFLYII | Membrane | 32 | 49 | 17 |  |
| 48 | Me-PET_87-104_ | PETNILLNVPLHGTILT | Membrane | 87 | 104 | 17 |  |
| 49 | Me-MWL_91-114_ | MWLSYFIASFRLFARTRSMWSFNP | Membrane | 91 | 114 | 23 |  |
| 50 | Me-ELV_137-159_ | ELVIGAVILRGHLRIAGHHLGRC | Membrane | 137 | 159 | 22 |  |
| 51 | Me-SRT_173-192_ | SRTLSYYKLGASQRVAGDSG | Membrane | 173 | 192 | 20 |  |
| 52 | Me-LPK_164-178_ | LPKEITVATSRTLSY | Membrane | 164 | 178 | 14 |  |
| 53 | o8-FLV_3-20_ | FLVFLGIITTVAAFHQEC | orf8 | 3 | 20 | 17 |  |
| 54 | o8-HFY_40-59_ | HFYSKWYIRVGARKSAPLIE | orf8 | 40 | 59 | 19 |  |
| 55 | o3-ATP_23-41_ | ATPSDFVRATATIPIQASL | orf3a | 23 | 41 | 18 |  |
| 56 | o3-LLA_52-67_ | LLAVFQSASKIITLKK | orf3a | 52 | 67 | 15 |  |
| 57 | o3-QSA_57-74_ | QSASKIITLKKRWQLALS | orf3a | 57 | 74 | 17 |  |
| 58 | o3-TLK_64-83_ | TLKKRWQLALSKGVHFVCN | orf3a | 64 | 83 | 19 |  |
| 59 | o3-VYF_112-128_ | VYFLQSINFVRIIMRLW | orf3a | 112 | 128 | 16 |  |

**Table S5.** Main features of peptides tested in the different peptide pools used in this study. Peptides are named after the antigen they belong to and the first three amino acids in the peptide followed by the position in the corresponding antigens. Bold characters in the peptide sequence column indicate amino acids showing variability over the strains indicated in the last column [52].

**References**

1. Tarke A, Sidney J, Kidd CK, Dan JM, Ramirez SI, Yu ED, et al. Comprehensive analysis of T cell immunodominance and immunoprevalence of SARS-CoV-2 epitopes in COVID-19 cases. Cell Rep Med. 2021;2:100204.

2. Karsten H, Cords L, Westphal T, Knapp M, Brehm TT, Hermanussen L, et al. High-resolution analysis of individual spike peptide-specific CD4+ T-cell responses in vaccine recipients and COVID-19 patients. Clin Transl Immunology.2022;11:e1410.

3. Heide J, Schulte S, Kohsar M, Brehm TT, Herrmann M, Karsten H, et al. Broadly directed SARS-CoV-2-specific CD4+ T cell response includes frequently detected peptide specificities within the membrane and nucleoprotein in patients with acute and resolved COVID-19. PLoS Pathog. 2021;17:e1009842.

4. Mateus J, Grifoni A, Tarke A, Sidney J, Ramirez SI, Dan JM, et al. Selective and cross-reactive SARS-CoV-2 T cell epitopes in unexposed humans. Science (1979). 2020; 370(6512):89-94.

5. Johansson AM, Malhotra U, Kim YG, Gomez R, Krist MP, Wald A, et al. Cross-reactive and mono-reactive SARS-CoV-2 CD4+ T cells in prepandemic and COVID-19 convalescent individuals. PLoS Pathog . 2021;17(12):e1010203.

6. Lang-Meli J, Luxenburger H, Wild K, Karl V, Oberhardt V, Salimi Alizei E, et al. SARS-CoV-2-specific T-cell epitope repertoire in convalescent and mRNA-vaccinated individuals. Nat Microbiol. 2022 7:675-79.

7. Verhagen J, van der Meijden ED, Lang V, Kremer AE, Völkl S, Mackensen A, et al. Human CD4+ T cells specific for dominant epitopes of SARS-CoV-2 Spike and Nucleocapsid proteins with therapeutic potential. Clin Exp Immunol. 2021;205:363–78.

8. Nelde A, Bilich T, Heitmann JS, Maringer Y, Salih HR, Roerden M, et al. SARS-CoV-2-derived peptides define heterologous and COVID-19-induced T cell recognition. Nat Immunol 2020;22:74–85.

9. Titov A, Shaykhutdinova R, Shcherbakova O V., Serdyuk Y V., Sheetikov SA, Zornikova K V., et al. Immunogenic epitope panel for accurate detection of non-cross-reactive T cell response to SARS-CoV-2. JCI Insight . 2022;7(9):e157699.

10. Low JS, Vaqueirinho D, Mele F, Foglierini M, Jerak J, Perotti M, et al. Clonal analysis of immunodominance and crossreactivity of the CD4 T cell response to SARS-CoV-2. Science (1979). 2021;372:1336–41.

11. Prakash S, Srivastava R, Coulon P-G, Dhanushkodi NR, Chentoufi AA, Tifrea DF, et al. Genome-Wide B Cell, CD4 + , and CD8 + T Cell Epitopes That Are Highly Conserved between Human and Animal Coronaviruses, Identified from SARS-CoV-2 as Targets for Preemptive Pan-Coronavirus Vaccines. J Immunol. 2021;206:2566–82.

12. Jin X, Ding Y, Sun S, Wang X, Zhou Z, Liu X, et al. Screening HLA-A-restricted T cell epitopes of SARS-CoV-2 and the induction of CD8+ T cell responses in HLA-A transgenic mice. Cell Mol Immunol. 2021;18:2588–608.

13. Woldemeskel BA, Garliss CC, Blankson JN. SARS-CoV-2 mRNA vaccines induce broad CD4+ T cell responses that recognize SARS-CoV-2 variants and HCoV-NL63. J Clin Invest. 2021;131(10):e149335.

14. Eggenhuizen PJ, Ng BH, Chang J, Fell AL, Cheong RMY, Wong WY, et al. BCG Vaccine Derived Peptides Induce SARS-CoV-2 T Cell Cross-Reactivity. Front Immunol. 2021; 12:692729.

15. Nelson RW, Chen Y, Venezia OL, Majerus RM, Shin DS, Carrington MN, et al. SARS-CoV-2 epitope–specific CD4+ memory T cell responses across COVID-19 disease severity and antibody durability. Sci Immunol. 2022;7:eabl9464.

16. Emmelot ME, Vos M, Boer MC, Rots NY, de Wit J, van Els CACM, et al. Omicron BA.1 Mutations in SARS-CoV-2 Spike Lead to Reduced T-Cell Response in Vaccinated and Convalescent Individuals. Viruses. 2022;14(7):1570.

17. Rammensee HG, Gouttefangeas C, Heidu S, Klein R, Preuß B, Walz JS, et al. Designing a SARS-CoV-2 T-Cell-Inducing Vaccine for High-Risk Patient Groups. Vaccines. 2021;9(5):428.

18. Bartolo L, Afroz S, Pan Y-G, Xu R, Williams L, Lin C-F, et al. SARS-CoV-2-specific T cells in unexposed adults display broad trafficking potential and cross-react with commensal antigens. Sci Immunol. 2022; 7(76):eabn3127.

19. Keller MD, Harris KM, Jensen-Wachspress MA, Kankate V V., Lang H, Lazarski CA, et al. SARS-CoV-2-specific T cells are rapidly expanded for therapeutic use and target conserved regions of the membrane protein. Blood. 2020;136:2905–17.

20. Lin J, Law R, Korosec CS, Zhou C, Koh WH, Ghaemi MS, et al. Longitudinal Assessment of SARS-CoV-2-Specific T Cell Cytokine-Producing Responses for 1 Year Reveals Persistence of Multicytokine Proliferative Responses, with Greater Immunity Associated with Disease Severity. J Virol. 2022; 96(13):e0050922.

21. Heitmann JS, Bilich T, Tandler C, Nelde A, Maringer Y, Marconato M, et al. A COVID-19 peptide vaccine for the induction of SARS-CoV-2 T cell immunity. Nature 2022 601:7894 . 2021;601:617–22.

22. Mahajan S, Kode V, Bhojak K, Karunakaran C, Lee K, Manoharan M, et al. Immunodominant T-cell epitopes from the SARS-CoV-2 spike antigen reveal robust pre-existing T-cell immunity in unexposed individuals. Sci Rep. 2021;11:13264.

23. De Castro M V., Santos KS, Apostolico JS, Fernandes ER, Almeida RR, Levin G, et al. Recurrence of COVID-19 associated with reduced T-cell responses in a monozygotic twin pair. Open Biol . 2022;12(2):210240.

24. Bilich T, Nelde A, Heitmann JS, Maringer Y, Roerden M, Bauer J, et al. T cell and antibody kinetics delineate SARS-CoV-2 peptides mediating long-Term immune responses in COVID-19 convalescent individuals. Sci Transl Med . 2021;13:7517.

25. Loyal L, Braun J, Henze L, Kruse B, Dingeldey M, Reimer U, et al. Cross-reactive CD4+ T cells enhance SARS-CoV-2 immune responses upon infection and vaccination. Science (1979). 2021; 374(6564):eabh1823.

26. Le Bert N, Tan AT, Kunasegaran K, Tham CYL, Hafezi M, Chia A, et al. SARS-CoV-2-specific T cell immunity in cases of COVID-19 and SARS, and uninfected controls. Nature. 2020;584(7821):457-462.

27. Lu X, Hosono Y, Nagae M, Ishizuka S, Ishikawa E, Motooka D, et al. Identification of conserved SARS-CoV-2 spike epitopes that expand public cTfh clonotypes in mild COVID-19 patients. J Exp Med;218(12):e20211327.

28. Piadel K, Haybatollahi A, Dalgleish AG, Smith PL. Selection and T-cell antigenicity of synthetic long peptides derived from SARS-CoV-2. J Gen Virol . 2022;103(1):001698.

29. Mudd PA, Minervina AA, Pogorelyy M V., Turner JS, Kim W, Kalaidina E, et al. SARS-CoV-2 mRNA vaccination elicits a robust and persistent T follicular helper cell response in humans. Cell. 2022;185:603-613.e15.

30. Poluektov Y, George M, Daftarian P, Delcommenne MC. Assessment of SARS-CoV-2 specific CD4(+) and CD8 (+) T cell responses using MHC class I and II tetramers. Vaccine. 2021;39(15):2110-2116. 31. Panikkar A, Lineburg KE, Raju J, Chew KY, Ambalathingal GR, Rehan S, et al. SARS-CoV-2-specific T cells generated for adoptive immunotherapy are capable of recognizing multiple SARS-CoV-2 variants. PLoS Pathog. 2022 ;18(2):e1010339.

32. Li L, Chen Q, Han X, Shen M, Hu C, Chen S, et al. T Cell Immunity Evaluation and Immunodominant Epitope T Cell Receptor Identification of Severe Acute Respiratory Syndrome Coronavirus 2 Spike Glycoprotein in COVID-19 Convalescent Patients. Front Cell Dev Biol. 2021;9:2880.

33. Zhang Y, Yang Z, Tang M, Li H, Tang T, Li G, et al. Three Specific Potential Epitopes That Could Be Recognized by T Cells of Convalescent COVID-19 Patients Were Identified From Spike Protein. Front Immunol. 2022;13:75.

34. Peng Y, Mentzer AJ, Liu G, Yao X, Yin Z, Dong D, et al. Broad and strong memory CD4+ and CD8+ T cells induced by SARS-CoV-2 in UK convalescent individuals following COVID-19. Nat Immunol. 2020;21(11):1336–45.

35. Wragg KM, Lee WS, Koutsakos M, Tan HX, Amarasena T, Reynaldi A, et al. Establishment and recall of SARS-CoV-2 spike epitope-specific CD4+ T cell memory. Nat Immunol. 2022 23(5):768–80.

36. Pogorelyy M V., Rosati E, Minervina AA, Mettelman RC, Scheffold A, Franke A, et al. Resolving SARS-CoV-2 CD4+ T cell specificity via reverse epitope discovery. Cell Rep Med. 2022;3:100697.

37. Ma Y, Liu F, Lin T, Chen L, Jiang A, Tian G, et al. Large-Scale Identification of T-Cell Epitopes Derived From Severe Acute Respiratory Syndrome Coronavirus 2 for the Development of Peptide Vaccines Against Coronavirus Disease. J Infect Dis. 2021;224(6):956–66.

38. Obermair FJ, Renoux F, Heer S, Lee CH, Cereghetti N, Loi M, et al. High-resolution profiling of MHC II peptide presentation capacity reveals SARS-CoV-2 CD4 T cell targets and mechanisms of immune escape. Sci Adv. 2022;8(17):eabl5394.

39. Zhao J, Wang L, Schank M, Dang X, Lu Z, Cao D, et al. SARS-CoV-2 specific memory T cell epitopes identified in COVID-19-recovered subjects. Virus Res. 2021;304:198508.

40. Dykema AG, Zhang B, Woldemeskel BA, Garliss CC, Cheung LS, Choudhury D, et al. Functional characterization of CD4+ T cell receptors crossreactive for SARS-CoV-2 and endemic coronaviruses. J Clin Invest . 2021;131(10):e146922.

41. Swadling L, Diniz MO, Schmidt NM, Amin OE, Chandran A, Shaw E, et al. Pre-existing polymerase-specific T cells expand in abortive seronegative SARS-CoV-2. Nature 2022; 601(7891):110–7.

42. Oberhardt V, Luxenburger H, Kemming J, Schulien I, Ciminski K, Giese S, et al. Rapid and stable mobilization of CD8+ T cells by SARS-CoV-2 mRNA vaccine. Nature 2021;597(7875):268–73.

43. de Silva TI, Liu G, Lindsey BB, Dong D, Moore SC, Hsu NS, et al. The impact of viral mutations on recognition by SARS-CoV-2 specific T cells. iScience. 2021;24:103353.

44. Schmidt KG, Nganou-Makamdop K, Tenbusch M, El Kenz B, Maier C, Lapuente D, et al. SARS-CoV-2-Seronegative Subjects Target CTL Epitopes in the SARS-CoV-2 Nucleoprotein Cross-Reactive to Common Cold Coronaviruses. Front Immunol. 2021;12:1029.

45. Hu W, He M, Wang X, Sun Q, Kuang M. Specific cd8+ tcr repertoire recognizing conserved antigens of sars-cov-2 in unexposed population: A prerequisite for broad-spectrum cd8+ t cell immunity. Vaccines (Basel) . 2021;9(10):1093.

46. Rowntree LC, Nguyen THO, Kedzierski L, Neeland MR, Petersen J, Crawford JC, et al. SARS-CoV-2-specific T cell memory with common TCRαβ motifs is established in unvaccinated children who seroconvert after infection. Immunity. 2022;55(7):1299-1315.e4.

47. Nagler A, Kalaora S, Barbolin C, Gangaev A, Ketelaars SLC, Alon M, et al. Identification of presented SARS-CoV-2 HLA class I and HLA class II peptides using HLA peptidomics. Cell Rep. 2021;35:109305.

48. Knierman MD, Lannan MB, Spindler LJ, McMillian CL, Konrad RJ, Siegel RW. The Human Leukocyte Antigen Class II Immunopeptidome of the SARS-CoV-2 Spike Glycoprotein. Cell Rep. 2020;33:108454.

49. Parker R, Partridge T, Wormald C, Kawahara R, Stalls V, Aggelakopoulou M, et al. Mapping the SARS-CoV-2 spike glycoprotein-derived peptidome presented by HLA class II on dendritic cells. Cell Rep. 2021;35:109179.

50. Liu R, Jiang W, Mellins ED. Yeast display of MHC-II enables rapid identification of peptide ligands from protein antigens (RIPPA). Cell Mol Immunol. 2021;18(8):1847–60.

51. Prachar M, Justesen S, Steen-Jensen DB, Thorgrimsen S, Jurgons E, Winther O, et al. Identification and validation of 174 COVID-19 vaccine candidate epitopes reveals low performance of common epitope prediction tools. Sci Rep. 2020 10(1):20465.

52. Bai H, Zhang X, Gong T, Ma J, Zhang P, Cai Z, et al. A systematic mutation analysis of 13 major SARS-CoV-2 variants. Virus Res; 2024;345:199392.
